# The Impact of Setting the Standards of Health Promoting Hospitals on Hospital Indicators in Iran

**DOI:** 10.1371/journal.pone.0167459

**Published:** 2016-12-13

**Authors:** Mohammad Amiri, Ahmad Khosravi, Leila Riyahi, Shima Naderi

**Affiliations:** 1 School of Public Health, Shahroud University of Medical Sciences, Shahroud, Iran; 2 Center for Health Related Social & Behavioral Sciences Research, Shahroud University of Medical Sciences, Shahroud, Iran; 3 Department of Health Services Management, Science and Research Branch, Islamic Azad University (IAU), Tehran, Iran; 4 Shahroud University of Medical Sciences, Shahroud, Iran; University of South Australia, AUSTRALIA

## Abstract

Hospitals play a critical role in the health promotion of the society. This study aimed to determine the impact of establishing standards of health promoting hospitals on hospital indicators in Shahroud. This applied study was a quasi-experimental one which was conducted in 2013. Standards of health promoting hospitals were established as an intervention procedure in the Fatemiyeh hospital. Parameters of health promoting hospitals were compared in intervention and control hospitals before and after of intervention (6 months). The data were analyzed using chi-square and t-test. With the establishment of standards for health promotion hospitals, standard scores in intervention and control hospitals were found to be 72.26 ± 4.1 and 16.26 ± 7.5, respectively. T-test showed a significant difference between the mean scores of the hospitals under study (P = 0.001).The chi-square test also showed a significant relationship between patient satisfaction before and after the intervention so that patients’ satisfaction was higher after the intervention (P = 0.001). Commenting on the short-term or long-term positive impacts of establishing standards of health promoting hospitals on all hospital indicators is a bit difficult but preliminary results show the positive impact of the implementation of standards in case hospitals which has led to the improvement of many indicators in the hospital.

## Introduction

The World Health Organization with the publication of "The Ottawa Charter for Health Promotion," in 1986 offered the first definition of health promotion services [1].Accordingly, health promotion encompasses concepts of health education, prevention of diseases, and rehabilitation services [2] and it includes an essential part of the continuum of treatment and clinical services [3]. Emphasis on prevention of diseases and health promotion services provided an opportunity for demanding and establishing health promotion services in hospitals. In the meantime, due to the dynamic role of hospitals in providing comprehensive health care services and benefits to patients, care takers, staff and the community, orienting clinical and therapeutic services towards the improvement of health services in hospitals seems necessary [1]. In addition to this, hospitals because of their central role in providing health services in the community and their interaction with different categories of patients, staff and organizations have great potentials to influence health promotion services and provide these services [4]. For this reason, it is necessary to change attitudes toward the role and capabilities of hospitals as promoting health structures [5]. Hospitals can promote public health through participation in health cycle [6]. However, at present, hospitals only serve their traditional roles in diagnosis and treatment and there is no defined structure for hospitals to provide many of the health promotion services [5] and the standards in the field of health care cannot lead to the appropriate development of health and health promotion in the community [7]. Going out of this situation requires new thinking in the field of health so that maximum utilization of existing facilities is made to provide and improve public health, and sustained and desirable results are achieved [5]. This will be achieved by establishing health promoting hospitals. The World Health Organization also encourage hospitals to adopt standards for optimum health promotion [1] and in defining health promoting hospital has stated that a health promoting hospital should go beyond providing high quality comprehensive medical and nursing services and developing a corporate identity that embraces participatory roles for patients and staff, seeks active links to cooperate with the community, and creates supportive environments for sustainable ecological development [8]. In health promoting hospitals, patients, their family members and health care providers have participatory roles in the decision-making process and health providing services. Values respected in health promoting hospitals include patient rights, employee rights, health equity, participation in decision-making and accountability [2]. In addition, health promoting hospitals focus on needs of patients and their companions [9], provide the circumstances for the creation of a healthy lifestyle for patients and the community, encourage the staff into healthy behavior and lifestyle and through reducing environmental risks try to promote the health of the staff [10].

In the Ninth International Conference on Health Promoting Hospitals in 2001, World Health Organization were developed five standards of HPH addressing the following issues:

1. Management policy: The organization has a written policy for health promotion at patients, relatives and staff. This policy aimed to improve health indicators.
2. Patient assessment: The organization ensures that there is a systematic need assessment in partnership with patients.
3. Patient information and intervention: The organization provides more concise information about the factors and conditions related to the patients. Health promotion interventions are established in all patient pathways.
4. Promoting a healthy workplace: The management provides a healthy environment by promoting of conditions in the organization.
5. Continuity and cooperation: There is an inter-sectional and intra-sectional collaboration with other health service levels and other institutions [11], which focus on four areas of patient health promotion, staff health promotion, changing the organization into a health promoting place and promoting the health of community [8].

Now with the development of an international network of health promoting hospitals in more than 40 countries, health promoting hospitals approach has been used in more than 800 hospitals [12]. However, the development of this network has been slow in developing countries and in spite of recent advances in the field of health care in these countries, the idea of health promotion has progressed slowly in their hospitals [4] and implementation of health promotion services has remained an unknown part in the transformation of the healthcare system in these countries [13]. Perhaps one reason for this is the lack of strategies, guidelines and tools on how to implement health promotion services [4]. The approach of health promoting hospitals has a short history in Iran and studies and experiences with a focus on health promotion are very limited in Iran. The implementation of this approach in Iranian hospital seems to be in the very early stages [8] and there is little evidence of the effectiveness of health promotion standards on health outcomes [12]. However, the results of some studies in Iran and the world suggest that the implementation of health promotion activities in hospitals has led to the improvement of the quality of health services [14,10], improvement of the clinical outcomes after treatment and improve effectiveness of health [15], increase in patient satisfaction, decrease in the length of patient stay in hospital [13,12,3], increase in the welfare of staff and patients [16], raising the awareness and information of patients [8], increase in job satisfaction and motivation of employees [13], improvement of the effectiveness and efficiency in hospitals [9], decrease in treatment complications, repeated admissions and cost of treatment [5] decrease in mortality [3], prevention of diseases, improvement in health indicators [13] and improvement of the quality of life of patients [17].Then, hospitals must design a specific system for improving and evaluating health promotion and therefore encourage policy-makers and health service administrators to invest resources in HPH [18]. Given the above, and given the importance and novelty of this topic, this study aimed to evaluate the impact of setting the standards of health promoting hospitals on hospital indicators.

## Material and Methods

This applied study was a quasi-experimental study conducted in 2013 in Shahroud, northeast of Iran. From 3 general hospitals of Shahroud, Fatemiyeh 96-bed hospital was randomly selected as the intervention and Khatamolanbia 96-bed hospital was selected as the control. In the first phase, selection of one hospital from 3 activated hospitals in the Shahroud city as an intervention hospital was based on simple random selection from 1, 2, 3.

This study and its' methodology were approved in research department of Science and Research Branch, Islamic Azad University, Tehran, Iran. Also, the permission for implementation of study was taken from research department of Shahroud University of Medical Sciences. The members of ethic committee in Shahroud University of Medical Sciences were checking the research proposal, health promoting hospital's questionnaire and satisfaction questionnaire. Also research methodology was explained detail in proposal and they were approved them. At admitting unit, all patients or her/his representative fill out a written informed consent form about permission to use medical information records.

Intervention: For the implementation of the standards of health promoting on the intervention hospital, the aim of study was explained to 170 staffs. The staffs were selected through stratified random sampling from 330 staffs who work at Fatemiyeh hospital. For selected staffs, conducted continuous educational programs. After the education of standard of health promoting, the selected staffs had cascade trainings for remaining staffs. With this method we reach to abroad training for establishment of intervention. The standards of health promoting hospitals established in March 2013. The implementation of intervention was carried out by an existing improving quality committee with cooperation of researcher and educational supervisor of the hospital.

Measurements: The standards of health promoting hospitals as intervention were established in Fatemiyeh hospital. For evaluation of impact of intervention, a number of indicators of health promoting hospitals for 6 months before and 6 months after intervention were compared in the intervention and control hospitals. Beside the comparison of the health indicators, we used a standard scale for evaluation of the hospitals. The health promoting hospital scale was used to evaluate standards of health promoting hospitals in the present study. The scale included 40 items on a 5 sections of management policies (9 items), patient assessment (7 items), patient information and intervention (6 items), creating a healthy work environment (10 items) and the continuity and cooperation (8 items).The items were three-choice ones and respondents needed to choose one of the three options of Yes (2 points), Partially (1 point) and No (0 point). Reliability and validity of the scale was confirmed by the Ministry of Health and Medical Education [19].The evaluation before and after the intervention was implemented by the hospital management team, quality improvement experts, training supervisors, clinical supervisors, supervisors, nutritionist, experts of medical records and environmental health expert in both hospitals.

With the establishment of health promotion standards in the intervention hospital and training of the staffs in line with the 5 sections of the program, measures such as patient and relatives education, assessment of nutritional status in hospitalized patients, patient assessment of risk factors, providing a safe and secure work environment and examination of occupational health the hospital staff began and indicators of health promoting hospitals were calculated after the intervention. In the indicator of assessing general risk factors for hospitalized patients (smoking, nutrition, psychosocial status, economic status, obesity and physical activity), a random sample of 40 patients were assessed.

Patient satisfaction was assessed in 8 areas (behavior of the medical team, on time attendance of the treatment team for clinical measures, respecting the privacy of patients during clinical measures, hospital hygiene and cleanliness, implementation of medical diagnostic methods, trainings offered to patients, welfare facilities, environment and quality of hospital food) in 80 random selected inpatients before and after intervention.

## Results

With the establishment of the standards of health promoting hospitals, the total scores of the intervention and control hospitals were 72.26±4.1 and 16.26±7.5, respectively. T-test showed a significant differences between the mean scores in the hospitals under study (P = 0.001) so that the mean score of the intervention hospital in each of the 5 standards increased after health intervention and they were significantly higher than those in the control hospital (Table 1).

**Table 1:** Mean scores of standards of health promoting hospitals in hospitals under study.

Standard area	Hospital	Mean±SD	t	P
**Management Policy**				
	intervention	1.35±14.6	—22.15	0.001
	Control	1.5 ±3		
**Patient Assessment**				
	intervention	0.9±13.4	—11.11	0.001
	Control	2.7±5.26		
**Patient Information and Intervention**				
	intervention	0.5±11.86	—13. 64	0.001
	Control	2.1±4.06		
**Promoting a Healthy Workplace**				
	intervention	1.6±17.6	—19.75	0.001
	Control	2.4±2.6		
**Continuity and cooperation**				
	intervention	1.9±14.8	—22.25	0.001
	Control	1.3±1.3		
**Total**				
	intervention	4.1±72.26	—25.29	0.001
	Control	7.5±16.26		

SD, standard deviation.

By setting standards and holding cascade trainings in the intervention hospital, all nurses, midwives and service personnel, 60% of doctors and 30% of administrative and support staff became aware of health promotion sections. In total, more than 85% of staff became aware of health promotion topics. In the indicator of assessing general risk factors for hospitalized patients no one smoked, 72.5% had optimal nutrition, 65% had a favorable psychosocial status, 32.5% had an appropriate economic situation, 7.5% were obese and 30% were taking regular physical exercise. Other calculated indices are shown in Table 2.

**Table 2:** Some hospital parameters before and after the intervention.

Parameter		Before Intervention		After Intervention	
		N	%	N	%
Admitting special patients in hospital					
	**Cancer patient**s	9	0.14	10	0.16
	**chronic obstructive pulmonary disease**	0	0	3	0.05
	**Patients stroke**	4	0.06	7	0.11
	**asthma patients**	2	0.03	6	0.10
	**Surgery patients**	1349	21.21	1337	21.95
**% of patients educated about specific actions inself-management of their condition** n = 40		10	25	33	82.5
**% of patients assessed for nutrition** n = 40		5	12.5	20	50
**% of work-related injuries**		1	0.49	0	0
**% of staff checkups about Occupational health**		0	0	330	100

The impact of establishment of health promoting standards in the intervention hospital, on patient satisfaction in 8 areas are summarized in Table 3.

**Table 3:** The impact of standards of health promotion on patient satisfaction.

Satisfaction area	Rating	Before Intervention		After Intervention		p.v
		N	%	N	%	
Behavior of the medical tea						
	Good	30	37.5	53	66.2	0.001
	Moderate	34	42.5	19	23.8	
	Poor	16	20	8	10	
On time attendance of the treatment team for clinical measures						
	Good	20	25	50	62.5	0.001
	Moderate	41	51.2	22	27.5	
	Poor	19	23.8	8	10	
Hospital hygiene and cleanliness						
	Good	34	42.5	49	61.2	0.018
	Moderate	33	41.2	27	33.8	
	Poor	13	16.2	4	5	
Implementation of medical diagnostic methods						
	Good	37	46.2	54	67.5	0.015
	Moderate	26	32.5	19	23.8	
	Poor	17	21.2	7	8.8	
Trainings offered to patients						
	Good	18	22.5	59	73.8	0.001
	Moderate	29	36.2	19	23.8	
	Poor	33	41.2	2	2.5	
Welfare facilities and environment						
	Good	8	10	20	25	0.001
	Moderate	19	23.8	52	65	
	Poor	53	66.2	8	10	
Food quality						
	Good	39	48.8	45	56.2	0.5
	Moderate	30	37.5	28	35	
	Poor	11	13.8	7	8.8	
Respecting the privacy of patients during clinical measures						
	Good	41	51.2	55	68.8	0.68
	Moderate	26	32.5	15	18.8	
	Poor	13	16.2	10	12.5	
General satisfaction						
	Good	2	2.5	39	48.8	0.001
	Moderate	67	83.8	41	51.2	
	Poor	11	13.8	0	0	

Chi-square test showed a significant relationship between patient satisfaction with the medical team behavior, on time attendance of the treatment team for of clinical measures, hospital hygiene and cleanliness, implementation of medical diagnostic methods, trainings offered to patients and welfare facilities and hospital environment and situation before and after the establishment of health promoting standards (P = 0.001) so that the percentage of satisfaction in the hospital was greater after the intervention. Chi-square test did not show a significant relationship between food quality and respecting the privacy of patients before and after the establishment of health promoting standards (P≥ 0.05). A significant relationship was also observed between patient satisfaction before and after the intervention so that overall satisfaction increased after the intervention. (P = 0.001).

The scores of the parameter of trainings offered to patients before and after the intervention are displayed in Table 4.

**Table 4:** Impact of the establishment of health promoting standards of hospitals on patient training.

Variable	Mean±SD before Intervention	Mean±SD after Intervention	p.v
On arrival training	1.5±0.97	2.8±0.49	0.001
During Hospitalization training	1.85±1.1	3.6±0.67	0.001
On discharge training	0.85±0.73	1.8±0.42	0.001

SD, standard deviation.

T-test showed a significant difference between the mean scores of patient training before and after the establishment of standards of health promoting hospitals (P = 0.001) so that the mean scores of the patients after the establishment of standards was prominently higher.

## Discussion

The results of the present study showed that total scores of the intervention hospital of the standards of health promoting hospitals was 72.26±4.1 (out of 80) While the hospital evaluated in the study of Lin et al.[20] obtained the score of 73.12 (out of 100) and the hospitals in the study of Groene et al. [6] achieved the score of 71.9± 25 (out of 136) and the score of the hospitals examined in the study by Yaghoubi et al. [7] was54.1 ± 15.1 (out of 136) and score of hospitals surveyed in the study of Lin et al.[10] was 76.26 (out of 100);therefore, the score of hospital surveyed in this study was higher than the hospitals in the above-mentioned studies. In the Lin study in Taiwan [10] a cross-sectional survey of all Taiwan’s hospitals above the local community hospital level was conducted. The questionnaire consisted of four subscales: policy and leadership, physical environment, healthy culture and health resource and activity. The standardized overall score of the organizational HPH status achieved was 76.26 (out of a possible score of 100). With respect to the subscales, physical environment scored the highest with 83.42; followed by healthy culture, scoring 78.93. This was followed by policy and leadership, with a score of 75.38, with the lowest score, 72.35, being obtained by health resource and activity.

Groene et al. [6] stated that hospitals which were evaluated with external assessments such as EFQM, Accreditation and ISO could improve in hospital processes and get better results in the establishment of standards for health promotion. The present study also implemented accreditation standards and the hospital was assessed through accreditation, which can perhaps be one reason for the high rating on standards for health promotion in hospitals and it is confirmed by the results of the study by Groene et al. [6]. Moreover, Groene et al. [6] stated that because of better economy, large hospitals (more than 400 beds) compared to smaller hospitals have a better opportunity to implement health promotion standards and will be successful in the process of development and implementation of health promotion structures and activities. They reported a significant relationship between the number of beds and average standard score (P = 0.008) so that the mean score of hospitals with more than 400 beds was significantly higher than that of hospitals with less than 400 beds. However, the hospital investigated in the present study was a small hospital with less than 100 beds but could achieve a high rating on the health promotion standards. This is not consistent with the results of Groene et al. [6].Perhaps in the smaller hospitals it is easier to connect with the staff and to exchange information between employees, and due to the lower number of hospitalized patients, the staffs have a better opportunity to interact with patients and see to their demands and therefore, the establishment and implementation of programs to improve the quality of the hospitals is more successful.

According to the obtained results, a significant difference was observed between the total mean scores of health promotion standards between two studied hospitals. The mean scores in all 5 areas of health promotion standards in the intervention hospital were significantly higher than those in the control hospital (Table 1). The highest score was obtained in the area of healthy work environment which is consistent with the results of Lin et al. [10]. Perhaps one of the reasons is the small size and limited space of the hospital which, despite the small number of staff and limited physical environment, made the implementation of health promotion standards easier. However, in the study of Lin et al. [20] and Yaghoubi et al. [7], the highest scores were in the standard of patient information and intervention.

In the study of Yaghoubi et al. [7] the lowest score obtained in the patient assessment area. While the lowest score in the present study was in patient information and intervention area, which is consistent with Groene et al. [6]. Perhaps one of the reasons for not obtaining a high score on this area is that health promotion activities for the patients are new, because patients’ and staff’s lacking of prior knowledge of this program, caused problems for the establishment and implementation of this area. It should also be considered that evaluation of patients for risk factors, their health promotion needs and taking measures to promote their health was done for the first time in the hospital and it is normal to have problems in the early stages.

In this study, none of the patients were smokers, but in the study conducted by Groene et al. [21]18.78% of patients were smokers, and in the research by Haynes [22] 80% of patients were smokers, in the study by Habibi sola et al. [23] 15% of patients were smokers and in the study of Oppedal et al. [24] 18% of patients were smokers. Therefore, the rate of smoking in this study was less than that in all the studies mentioned above.

The assessment also showed that in the intervention hospital patients, only 30% of the sample had regular physical activity, while in Groene et al. [21] 84% of patients had regular physical activity; in the study by Haynes [22] 39% of the patients had regular physical activity and in the study by Habibisola et al. [23] 64.6% of patients had regular physical activity, so the patients in the current study had the least physical activity. In assessing obesity in the sample, 7.5% of patients were obese while in the study by Haynes [22]86% of patients and in the study of Oppedal et al. [24] 68% of the patients were overweight. The results show that obesity of the patients in the study was less than the studies conducted in other hospitals.

The assessment of nutritional status in Fatemiyeh hospital (intervention hospital) showed that 72.5 patients were in good nutritional status, while in Groene et al. study [21] 75% of patients had good nutritional status and in Oppedal et al. study [24], 56% of patients had a good nutrition. So, the percentage of patients with good nutritional status in the intervention hospital was less than that in the study of Groene et al.[21] and more than that in the by Oppedalet.al., they evaluated several risk factors such as overweight, under-nutrition, physical inactivity and smoking. Results showed that 68% of patients were overweight, 44% at risk of under-nutrition, 38% physically inactive and 19% were daily smokers. In totally 91% of the patients had at least one health risk factor. As many as 58% had two or more risk factor, 19% had three or more, 3% had four or more and three patients had all five risk factors. The multivariate analysis showed that having more than one health risk factor was associated with hospitalization [24].

With the establishment of standards for health promotion in hospitals, 93% of employees in Fatemiyeh hospital stated when necessary, they cooperated with other relevant organizations such as the Association of Special Patients, Cancer Society, Rehabilitation Services, Welfare organization, etc., Also in another study, the community health activity was at the higher level in the health promotion dimension, it showed the importance of community activities. Community activities were related to improve self-care, management of chronic illness and life style development. In fact, improvement of community health leads to health promotion of hospital [18].

In the study by Polluste et al. 72% of managers in the international network of health promoting hospitals and 33% of managers of the hospitals which were not members of the network said that when necessary, they cooperated with other relevant organizations. So, the results showed that cooperation with other relevant organizations was higher in the case hospital compared to the hospitals studied in the research Polluste et al. [14].The results of a similar study showed that cooperating with relevant agencies and follow-up of patients after discharge from hospital is among the affordable ways to improve the patients ‘quality of life [17]. Hospitals are going to realize their socio-political and health policy potential. They must firstly realize it, want it and earn it. Also one of the important of Vienna recommendations for Health Promoting Hospitals expressed as expanding the hospitals’ public health role, in alliance with the population of the local community and its social and health services, thus optimizing links between different providers, users and actors in the ‘whole’ health and social care sector [25].

In summary, health promotion is a process in which the patients can learn how to take care of themselves through community participation and the development of the organization that demonstrates to patients and relatives ways to adjust their behaviors after going back to their homes. Caring for theme selves after going back home was well-being behavior that results from the health promoting process. Community participation can help by extending the self-care process among the community [26].

In the study after the intervention, 100% of the Nursing and Midwifery staff, 60% of physicians and 30% of administrative staff were aware of standards of health promotion program in hospitals, in another study staff trained completely and they played important role in establishment of HPH' strategies, the staff of the hospital engaged in range of health promotion activities impacted not only on the health of patients and their families, but on staff, the organization, the physical environment and the broader community [27]. While in the study of Polluste et al. [14], 98% of nurses, 86% of physicians and 74% of cleric staff were aware of health promotion programs and in the study of Miseviciene et al. [12], only 35.8% of employees were aware of the health promotion goals and activities. Taking these studies into account it can be said that the nurses in the case hospital were more aware of the health promotion program than those in Polluste et al., while the percentage of physicians and administrative staff who were aware of health promotion programs in this study was less than those in Polluste et al. [14]. It should be noted that in the implementation and establishment of the modern programs of hospital quality improvement, nurses have a more important role than other health care groups and because of their interaction with patients, they bear the greatest burden of the program and consequently their awareness of programs becomes more than other groups.

In this study, 82.5% of patients acknowledged that they received the necessary trainings on disease management and the average rating of on admission trainings, during hospitalization and after discharge, was significantly higher after intervention, while in the study by Haynes [22] only one-third of the patients admitted that they have received trainings necessary for their disease. In the same study patient empowered in self-care, participation in treatment, also they empowered in management of chronic illness and lifestyle development. Patient health promotion had a good score in dimensions of Health Promotion [18].

After intervention for health promotion in hospitals, occupational health examinations were conducted for all employees while in the study by Miseviciene et al. [12], only 88.6% of the personnel underwent annual occupational health examinations. Currently, globally an estimated two million people die each year as a result of occupational accidents and work-related illnesses or injuries. Healthy work places envision building a healthy workforce as well as providing them with healthy working conditions. Healthy working environments translate to better health outcomes for the employees and better business outcomes for the organizations [28].

In this study, patient satisfaction increased after the intervention which is consistent with the results of Khowaja et al. [13] and Polluste et al. [14]. Settlement the quality improvement teams (QIT) in hospitals is an important step for total quality management (TQM) implantation, and is a component of participatory management mechanism.

Findings of the study about implementation of quality improvement program proved that the rate of patient satisfaction have improved in the following subjects: imaging services, employee behavior, cleaning, food quality cleaning of wards, environment peacefulness of wards, availability of drugs, administrative supervision, social workers performance and quick discharge activities [29].

## Conclusion

In this study commenting on the short-term or long-term positive impacts of establishing standards of health promoting hospitals on all hospital parameters is a bit difficult but preliminary results show the positive impact of the implementation of standards in intervention hospital which has led to the improvement of many parameters in intervention hospital. Therefore, in order to improve health promotion activities in hospitals across the country and to increase the members of international network of health promoting hospitals (HPH), the establishment of this program in all government and private hospitals is suggested.

As well as from this research, recommendations that could help and improve health promoting hospitals are given below:

1. In order to understand what people really need, the study of health promotion development should include the case studies of the serviced users (patients) who receive the health promotion services from the hospitals.
2. The policy in health promotion should be clear.
3. The setting and planning of the participation in hospitals should be more visible and comprehensible. This research found that there were various activities, but only hospital staffs, people in the communities, or health promotion volunteers were usually in charge of the activities but there was no participation from other organizations.
4. The health promoting activities should be reviewed after they are completed in order to improve them and make them better.
5. The development of the health promoting networks, such as the health promotion volunteers and other volunteer groups, should have visible roles in order to make health promotion more effective.

## Supporting Information

S1 Checklist Standards for Health Promotion in Hospitals. Self-assessment tool for pilot implementation.
(DOCX)

S1 Dataset Assessment of health promoting hospitals’ standards. (SAV)

S2 Dataset The data of patient satisfaction before and after intervention. (SAV)

S3 Dataset The data of patient training before and after intervention. (SAV)

S1 Protocol Implementation health promotion in hospitals: manual and self-assessment forms. (PDF)
