# Supplementary material for: The Impact of Setting the Standards of Health Promoting Hospitals on Hospital Indicators in Iran
Source: PLoS One. 2016 Dec 13;11(12):e0167459. doi: 10.1371/journal.pone.0167459 (PMC5154508; doi:10.1371/journal.pone.0167459)
Supplement: S1 Checklist — Self-assessment tool for pilot implementation. (DOCX) [file pone.0167459.s001.docx]

| Number | **Management Policy** | Yes | Partly | No |
| --- | --- | --- | --- | --- |
| 1.1 | The hospital’s stated aims and mission include health promotion [Evidence: e.g. time- table for the action]. |  |  |  |
| 1.2 | Minutes of the governing body reaffirm agreement within the past year to participate in the WHO HPH project [Evidence: e.g. date for the decision or for payment of the annual fee]. |  |  |  |
| 1.3 | The hospital’s current quality and business plans include health promotion (HP) for patients, staff and the community [Evidence: e.g. health promotion is explicit in the plan of action]. |  |  |  |
| 1.4 | The hospital identifies personnel and functions for the coordination of HP [Evidence: e.g. staff member nominated for the coordination of HP]. |  |  |  |
| 1.5 | There is an identifiable budget for HP services and materials [Evidence: e.g. budget or staff resources]. |  |  |  |
| 1.6 | Operational procedures such as clinical practice guidelines or pathways incorporating HP actions are available in clinical departments [Evidence: e.g. check guidelines]. |  |  |  |
| 1.7 | Specific structures and facilities required for health promotion (including resources, space, and equipment) can be identified [Evidence: e.g. facilities to lift patients available]. |  |  |  |
| 1.8 | Data are routinely captured on HP interventions and available to staff for evaluation [Evidence: e.g. availability assessed in staff survey]. |  |  |  |
| 1.9 | A programme for quality assessment of the health promoting activities is established [Evidence: e.g. time schedule for surveys is available]. |  |  |  |
| **Patient Assessment** | | Yes | Partly | No |
| 2.1 | Guidelines on how to identify smoking status, alcohol consumption, nutritional status, psycho social economic status are present [Evidence: e.g. check availability]. |  |  |  |
| 2.2 | Guidelines/procedures have been revised within the last year [Evidence: e.g. check date, person responsible for revising guidelines]. |  |  |  |
| 2.3 | Guidelines are present on how to identify needs for HP for groups of patients (e.g. asthma patients, diabetes patients, chronic obstructive pulmonary disease, surgery, rehabilitation) [Evidence: e.g. for groups of patients specifically treated in the clinical department]. |  |  |  |
| 2.4 | The assessment is documented in the patient’s record at admission [Evidence: e.g. identified by patient records audit]. |  |  |  |
| 2.5 | There are guidelines / procedures for reassessing needs at discharge or end of a given intervention [Evidence: e.g. guidelines present]. |  |  |  |
| 2.6 | Information from referring physician or other relevant sources is available in the patient’s record  [Evidence: for all patients referred from physician]. |  |  |  |
| 2.7 | The patient’s record documents social and cultural background as appropriate [Evidence: religion that requires special diet or other specific attention. Social conditions indicating that the patient is at risk]. |  |  |  |
| **Patient Information and Intervention** | | Yes | Partly | No |
| 3.1 | Information given to the patient is recorded in the patient’s record [Evidence: e.g. random review of patient records for all patients]. |  |  |  |
| 3.2 | Health promotion activities and expected results are documented and evaluated in the records [Evidence: e.g. patient records’ audit] |  |  |  |
| 3.3 | Patient satisfaction assessment of the information given is performed and the results are integrated into the quality management system [Evidence: e.g. various assessment methods: survey, focused group interview, questionnaire. Time schedule]. |  |  |  |
| 3.4 | General health information is available [Evidence: e.g. availability of printed or online information, or special information desk]. |  |  |  |
| 3.5 | Detailed information about high/risk diseases is available [Evidence: e.g. availability of printed or online information, or special information desk]. |  |  |  |
| 3.6 | Information is available on patient organizations [Evidence: e.g. contact-address is provided]. |  |  |  |
| **Promoting a Healthy Workplace** | | Yes | Partly | No |
| 4.1 | Working conditions comply with national/regional directives and indicators [Evidence: e.g. national and international (EU) regulations are recognized]. |  |  |  |
| 4.2 | Staff complies with health and safety requirements and all workplace risks are identified [Evidence: e.g. check data on occupational injuries]. |  |  |  |
| 4.3 | New staff receive an induction training that addresses the hospital’s health promotion policy [Evidence: e.g. interviews with new staff]. |  |  |  |
| 4.4 | Staff in all departments are aware of the content of the organization’s health promotion policy [Evidence: e.g. annual performance evaluation or staff participation in the HP programme]. |  |  |  |
| 4.5 | A performance appraisal system and continuing professional development including health promotion exists [Evidence: e.g. documented by review of staff files or interview]. |  |  |  |
| 4.6 | Working practices (procedures and guidelines) are developed by multidisciplinary teams [Evidence: e.g. check procedures, check with staff]. |  |  |  |
| 4.7 | Staff is involved in hospital policy-making, audit and review [Evidence: check with staff; check minutes of working groups for participation of staff representatives]. |  |  |  |
| 4.8 | Policies for awareness on health issues are available for staff [Evidence: e.g. check for policies on smoking, alcohol, substance misuse and physical activity]. |  |  |  |
| 4.9 | Smoking cessation programmes are offered [e.g. Evidence on availability of programmes]. |  |  |  |
| 4.10 | Annual staff surveys are carried out including an assessment of individual behavior, knowledge on supportive services/policies, and use of supportive seminars [Evidence: check questionnaire used for and results of staff survey]. |  |  |  |
| **Continuity and Cooperation** | | Yes | Partly | No |
| 5.1 | The management board is taking into account the regional health policy plan [Evidence: e.g. regulations and provisions identified and commented in minutes of the meeting of management board]. |  |  |  |
| 5.2 | The management board can provide a list of health and social care providers working in partnership with the hospital [Evidence: e.g. check update of list]. |  |  |  |
| 5.3 | The intra- and intersectoral collaboration with others is based on execution of the regional health policy plan [Evidence: e.g. check congruency]. |  |  |  |
| 5.4 | There is a written plan for collaboration with partners to improve the patients’ continuity of care [Evidence: e.g. criteria for admittance, plan for discharge]. |  |  |  |
| 5.5 | Patients (and their families as appropriate) are given understandable follow-up instructions at out-patient consultation, referral or discharge [Evidence: e.g. patients’ evaluation assessed in patient surveys]. |  |  |  |
| 5.6 | There is an agreed upon procedure for information exchange practices between organizations for all relevant patient information [Evidence: e.g. check availability of procedure]. |  |  |  |
| 5.7 | The receiving organization is given in timely manner a written summary of the patient’s condition and health needs, and interventions provided by the referring organization [Evidence: e.g. availability of copy]. |  |  |  |
| 5.8 | If appropriate, a plan for rehabilitation describing the role of the organization and the cooperating partners is documented in the patient’s record [Evidence: e.g. review of records]. |  |  |  |
