# Supplementary material for: The Impact of Setting the Standards of Health Promoting Hospitals on Hospital Indicators in Iran
Source: PLoS One. 2016 Dec 13;11(12):e0167459. doi: 10.1371/journal.pone.0167459 (PMC5154508; doi:10.1371/journal.pone.0167459)
Supplement: S1 Protocol — (PDF) [file pone.0167459.s005.pdf]

EUROPE

Implementing health promotion in hospitals:

Manual and self-assessment forms

Edited by:
Oliver Groene

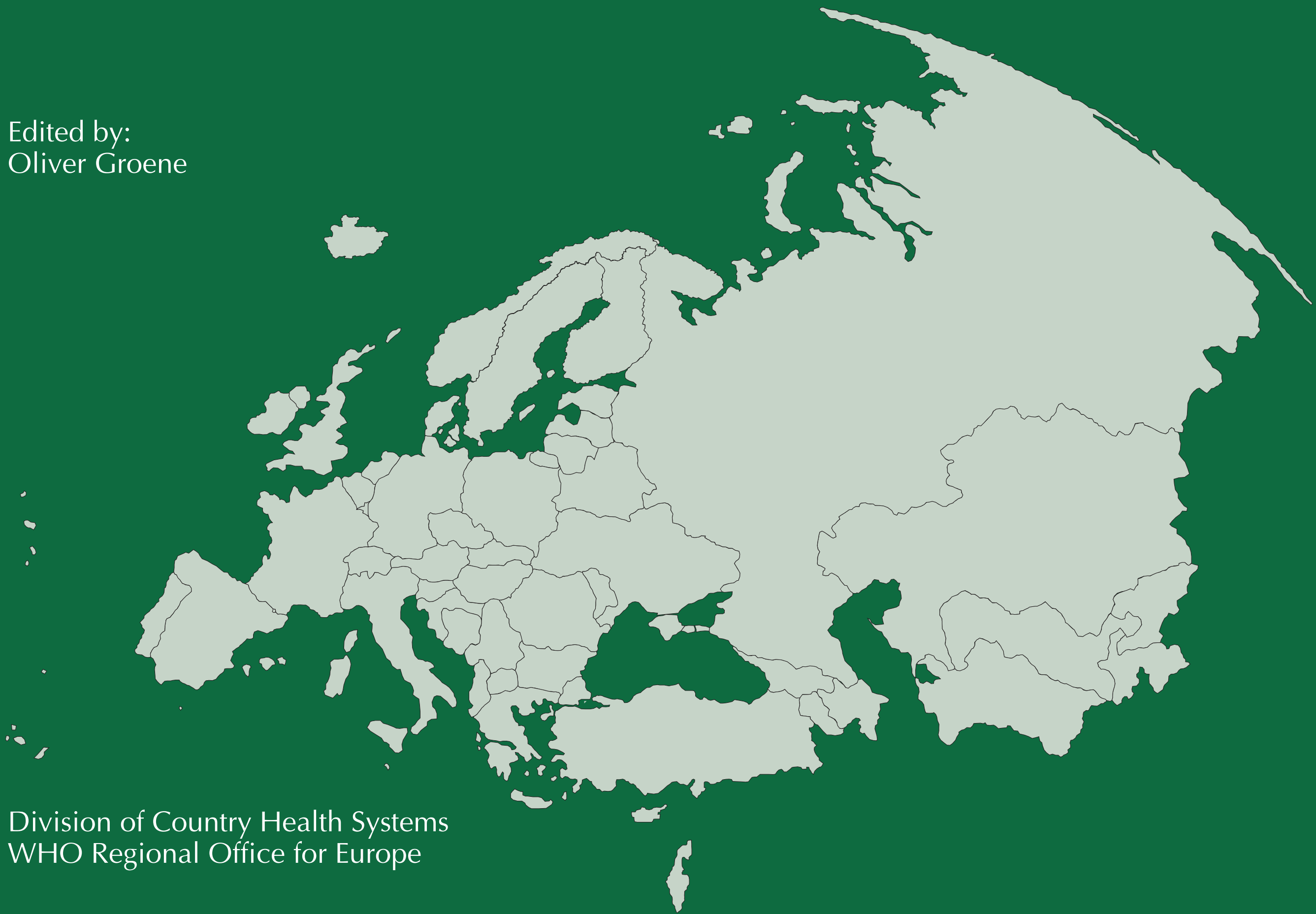

Division of Country Health Systems
WHO Regional Office for Europe

WORLD HEALTH ORGANIZATION
EUROPE

Implementing health promotion in hospitals:

Manual and self-assessment forms

Edited by:
Oliver Groene
Technical Officer, Quality of Health Systems and Services

Division of Country Health Systems
Country Policies, Systems and Services Unit
Barcelona Office

ABSTRACT

Health promotion is an integral part of the health care process and is related to clinical, educational, behavioural, and organizational issues. Quality improvement needs to embrace health promotion activities in order to make sure that effective approaches are used and continuously being monitored to improve outcomes. As many common quality management tools do not address health promotion activities explicitly, we developed this *"Manual on implementing health promotion in hospitals".* Its aim is to enable managers and health professionals to: assess health promotion activities in hospitals; improve the capacity of health care organizations in improving health promotion activities; formulate recommendations for the improvement of health promotion activities in hospitals; involve all professionals and the patient in improving the quality of care; improve the coordination of care with other providers of care; improve the health and safety of staff and patients. Individual hospitals, quality agencies and in particular members of the International Network of Health Promoting Hospitals are encouraged to use this tool and to assess and improve the quality of health promotion activities in health care.

Keywords

HOSPITALS
HEALTH PROMOTION - Standards
QUALITY INDICATORS
HEALTH CARE
SELF-EVALUATION PROGRAMS
EUROPE
EUR/06/5064214

Address requests about publications of the WHO Regional Office for Europe to:
Publications
WHO Regional Office for Europe
Scherfigsvej 8
DK-2100 Copenhagen Ø, Denmark
Alternatively, complete an online request form for documentation, health information, or for permission to quote or translate, on the WHO/Europe web site at http://www.euro.who.int/pubrequest.

© World Health Organization 2006

All rights reserved. The Regional Office for Europe of the World Health Organization welcomes requests for permission to reproduce or translate its publications, in part or in full.

The designations employed and the presentation of the material in this publication do not imply the expression of any opinion whatsoever on the part of the World Health Organization concerning the legal status of any country, territory, city or area or of its authorities, or concerning the delimitation of its frontiers or boundaries. Where the designation "country or area" appears in the headings of tables, it covers countries, territories, cities, or areas. Dotted lines on maps represent approximate border lines for which there may not yet be full agreement.

The mention of specific companies or of certain manufacturers' products does not imply that they are endorsed or recommended by the World Health Organization in preference to others of a similar nature that are not mentioned. Errors and omissions excepted, the names of proprietary products are distinguished by initial capital letters.

The World Health Organization does not warrant that the information contained in this publication is complete and correct and shall not be liable for any damages incurred as a result of its use. The views expressed by authors or editors do not necessarily represent the decisions or the stated policy of the World Health Organization.

Contents

	pages
Acknowledgements	5
List of figures and tables	6
1. Introduction	7
2. Frequently asked questions	9
3. Background and methodological issues	11
3.1. The role of hospitals in health promotion	11
3.2. Conceptualization of health promotion	12
3.3. Internal and external quality assessment	13
3.4. Standards for health promotion	15
3.5. Indicators for health promotion	19
3.6. Complementary use of standards and indicators	22
4. Project implementation	24
4.1. Clarifying responsibilities	24
4.2. Collecting data	25
4.3. Interpreting results	26
4.4. Developing a quality improvement plan	27
5. Assessment forms	29
Standard 1: Management Policy	33
Standard 2: Patient Assessment	38
Standard 3: Patient Information and Intervention	43
Standard 4: Promoting a Healthy Workplace	47
Standard 5: Continuity and cooperation	52
6. Descriptive sheets for indicators	59
7. Glossary	80

Acknowledgements

The development of this document has benefitted from the inputs of many people.
Thanks are due to all those who have supported and guided this work both within the working groups of the International Network of Health Promoting Hospitals and as external advisors.
Particular thanks are due to the country coordinators that supported the pilot test of this tool and to the health professionals in hospitals that took their time to assess compliance with standards and collected data on health promotion indicators.
Finally, Dr Svend Juul Jorgensen should be mentioned for his vision in respect of this project, Dr Mila Garcia-Barbero for her continuous support and Dr Kiki Lomberts for her expert advice on the final document.

List of figures and tables

Figure 1:	Strategies for health promotion	page 12
Figure 2:	Clinical, patients' and management perspective	page 16
Figure 3:	Three level structure of standards for health promotion	page 18
Figure 4:	Plan-Do-Check-Act Cycle	page 27
Table 1:	Overview of health promotion indicators	page 21
Table 2:	Descriptive sheet for staff awareness of policy	page 62
Table 3:	Descriptive sheet for patients' (and relatives') awareness	page 63
Table 4:	Descriptive sheet for percentage of health promotion budget	page 64
Table 5:	Descriptive sheet for patients assessed for generic risk factors	page 65
Table 6:	Descriptive sheet for patients assessed for specific risk factors	page 66
Table 7:	Descriptive sheet for patient satisfaction	page 67
Table 8:	Descriptive sheet for patient education in self-management	page 68
Table 9:	Descriptive sheet for patient risk factor education	page 69
Table 10:	Descriptive sheet for patients' information/intervention score	page 70
Table 11:	Descriptive sheet for staff smoking	page 71
Table 12:	Descriptive sheet for smoking cessation	page 72
Table 13:	Descriptive sheet for staff experience	page 73
Table 14:	Descriptive sheet for short-term absenteeism	page 74
Table 15:	Descriptive sheet for work-related injuries	page 75
Table 16:	Descriptive sheet for burnout scale	page 76
Table 17:	Descriptive sheet for discharge summaries	page 77
Table 18:	Descriptive sheet for readmission rate	page 78
Table 19:	Descriptive sheet for discharge preparation	page 79

1. Introduction

The role of health promotion in hospitals is changing. It is no longer restricted to providing additional lifestyle-related information to the patient after the clinical procedures have been completed. Health promotion is becoming an integral part of the health care process and is related to clinical, educational, behavioural, and organizational issues[1]. In order to improve the quality of care for patients with chronic diseases and long-term conditions, health promotion activities in hospitals need to become better embedded in the larger health systems framework. With the expanded scope of health promotion activities, questions are raised regarding the quality assessment and improvement of these activities.

Among the prominent tools to improve quality in health care, such as professionally consented guidelines, standards and performance indicators, there is little focus on health promotion issues. We therefore developed a self-assessment tool for health promotion in hospitals that addresses the following issues: the hospitals' management policy; the patients' assessment with regard to risk factors and health needs, patients' health promotion information and -intervention; promoting a healthy workplace and continuity and cooperation of the hospital with other health, social and informal care providers[2].

This document provides a self-standing tool to assess, monitor and improve health promotion activities in hospitals. It is based on two complementary approaches of quality assessment: ***standards***, expressing professionally consented statements on health care structures or processes that should be in place and ***indicators***, addressing health care processes and outcomes and providing a quantitative tool to assess variations in performance over time or between institutions. In detail, this document should facilitate: assessing health promotion activities in hospitals; developing the capacity of health care organizations in improving health promotion activities; formulating recommendations for the improvement of health promotion activities in hospitals; involving all professionals and the patient in improving health promotion activities; improving the coordination of care with other providers of care;

1. Groene O, Garcia-Barbero M. eds. Health promotion in hospitals. Evidence and quality management. Copenhagen, WHO Regional Office for Europe, 2005 (http://www.euro.who.int/document/E86220.pdf, accessed 08 May 2006).
2. Standards for Health Promotion in Hospitals. Copenhagen, WHO Regional Office for Europe, 2003 (http://www.euro.who.int/document/e82490.pdf, accessed 08 may 2006).

improving the health and safety of staff and patients; assisting with modernizing and changing healthcare practice and service delivery to make it more efficient and effective.

The following section of this document provides a set of frequently-asked questions and answers regarding the design and application of this tool (Section 2). Subsequently, the background and methodology applied in the development of this tool is described (Section 3). The document also addresses practical issues of project implementation: how to carry out the self-assessment, how to identify data sources, interpret results and translate them into a quality improvement plan (Section 4). To support data collection, sections 5 and 6 include a set of assessment forms that can be used to assess compliance with standards (Section 5) and descriptive sheets for health promotion indicators (Section 6). The last section includes a glossary of terms on quality and health promotion issues (Section 7).

Health promotion covers conceptually a broad range of activities, interventions, methods and approaches, some of which were too broad for the scope of this document. A decision was taken to address in this self-assessment tool only those issues that are most easily recognized by professionals working with patients, and for which the strongest evidence base is available. Consequently some health promotion activities that were included in previous guiding documents of the Health Promoting Hospitals' Network are not fully reflected[3,4]. A comprehensive framework to guide strategic implementation of health promotion in hospitals and to guide the further development of health promotion standards is summarized in the Eighteen Core Strategies for Health Promotion in Hospitals[5]. Some of the standards (like patient assessment or information and intervention) are directly linked to patient safety issues[6]; however, this document provides additional tools for a wider approach to empower patients and staff and to complement existing quality and safety activities.

This document was developed for all hospitals and quality agencies interested in improving health promotion. Organizations working in the field of quality improvement are encouraged to review and incorporate the standards and indicators for health promotion in hospitals into their existing systems.

3. Ottawa Charter for Health Promotion. Geneva, World Health Organization, 1986. (http://www.who.int/hpr/NPH/docs/ottawa_charter_hp.pdf, accessed 08 May 2006).
4. The Vienna Recommendations on Health Promoting Hospitals. Copenhagen, WHO Regional Office for Europe, 1997 (http://www.euro.who.int/healthpromohosp/publications/20020227_1, accessed 08 May 2006)
5. Pelikan J, Dietscher C, Krajic K , Nowak P. Eighteen core strategies for Health Promoting Hospitals. In: Groene O, Garcia-Barbero M, eds: Health promotion in hospitals. Evidence and quality management. Copenhagen, WHO Regional Office for Europe, 2005: 48-67. (http://www.euro.who.int/document/E86220.pdf, accessed 08 May 2006).
6. Forward Programme 2005. World Alliance for Patient Safety. Geneva, World Health Organization, 2004. (http://www.who.int/patientsafety/en/brochure_final.pdf , accessed 08 May 2006)

2. Frequently asked questions

Q1 Is it compulsory for members of the WHO Health Promoting Hospitals Network (HPH) to undertake self-assessment?

No – at this stage the self-assessment is a voluntary evaluation. The tool is a service to the member hospitals to facilitate the identification of areas where improvement can be achieved.

Q2 What are the benefits for my hospital to perform this self-assessment?

Hospitals may perform self-assessment in order to improve patient care, patients' quality of life and health of staff. The self-assessment tool helps to identify quality gaps in health promotion services and supports the development of an action plan.

Q3 How does this fit in with other quality initiatives?

The process of setting standards is an integral part of continuous quality improvement. The health promotion standards developed in this manual aim to complement existing quality standards that do not have a concrete focus on health promotion. Complementary indicators have been added to allow quantitative assessment of performance over time. It is highly recommended to link the self-assessment of standards for health promotion to the quality strategies already in use in the hospital.

Q4 How does this contribute to patient safety?

Information, education and communication in health care are core elements in ensuring patient safey. This applies to both staff and patients, by increasing their level of awareness, motivation and responsiveness. For example, informed patients can play an important role in identifying risks and devising solutions during their hospital stay and after discharge. Informed staff will contribute to develop and maintain a healthy and safe workplace.

Q5 What is a standard?

In this document, standards describe the required level of achievement. This document defines five standards, each addressing a health promotion dimension. Each standard is divided into substandards, which are then split into a number of measurable elements.

Q6 How do we measure compliance with standards?

Compliance with standards is measured as a sum of fulfilling measurable elements and substandards. Measurable elements need to be assessed as being fully, partially or not fulfilled.

Q7 How should we interpret compliance with standards?

Compliance with standards identifies areas of good health promotion practice that you may want to use as an example elsewhere in your hospital. Non-compliance tells you where there is room for improvement.

Q8 What are indicators?

While the standards address a required level of achievement assessed as being partly, fully or not in place, for the purpose of this document we understand indicators to be quantitative tools addressing process and outcome domains of quality. They are typically described in terms of numerator and denominator.

Q9 How are standards and indicators related?

The indicators listed in this document are not used to assess compliance with the five standards. They rather address complementary issues that should be taken into consideration in monitoring, evaluating and improving the health promotion area assessed. Indicators are numerical expressions used to flag or screen areas for improvement.

Q10 How do we measure indicators?

Indicators need to be measured repeatedly over time in order to reflect continuous quality improvement. The manual specifies for each indicator its rationale, description of numerator and denominator, data source and stratification.

Q11 How can we build an action plan?

The action plan should be developed based on the assessment of standards, indicators and the comments and observations that have been added during the self-assessment process. The action plan should also address the main gaps identified during the assessment and reflect organizational priorities.

Q12 What happens to our action plan?

In order to ensure implementation and monitoring the action plan needs to be presented to executive management and included into the hospital's quality management system.

Q13 Will the tool be used for benchmarking with other hospitals?

At this stage, the tool is intended to be used for self-assessment only. WHO and the HPH Network Coordinators are considering the use of the tool for benchmarking in the future.

Q14 Will we get a certificate?

No, WHO will not issue certificates. Self-assessment, continuous quality improvement and the development of action plans will not result in a 'pass' or 'fail'. Each hospital is different and therefore will have a different set of tailor-made action plans developed. However, the International Network of Health Promoting Hospitals may decide in the future to develop a system to recognize the level of achievement in line with the standards and indicators presented in this document.

3. Background and methodological issues

3.1. The role of hospitals in health promotion

In the early 1990s WHO initiated an international initiative to support hospitals in engaging in health promotion. Hospitals joining the International Network of Health Promoting Hospitals aim to provide high quality comprehensive medical and nursing services by introducing health promotion activities for patients, staff and the community into their corporate identity and routine practice[7].

There is a large scope and public health motivation for providing health promotion strategies in health care settings[8]. Hospitals consume between 40% and 70% of the national health care budget and typically employ about 1% to 3% of the working population[9]. Hospitals as working places are characterized by a range of physical, chemical, biological and psychosocial risk factors. Paradoxically, in hospitals – organizations that aim to restore health – the acknowledgement of factors that endanger the health of their staff is poorly developed, despite strong evidence on the relationships between staff health, productivity and quality of patient care[10].

Furthermore, hospitals can have a lasting impact on influencing the behaviour of patients and relatives, who are more responsive to health advice in situations of experienced ill-health[11]. Given the increasing prevalence of chronic disease in Europe and throughout the world and low compliance with treatment, therapeutic education is becoming a major issue[12]. Many hospital treatments do not cure but rather aim at improving the quality of life of patients. To maintain this quality, patients and relatives have to be educated and more intensively prepared for discharge. While the main responsibility of the hospital for patient care ends with the discharge procedure, it is important to stress that from a health system perspective a high number of readmissions or complications could be prevented, if patients were better prepared and subsequent providers of medical and social care were kept involved.

7. Health Promotion Glossary. Geneva, World Health Organization, 1998 (http://www.who.int/hpr/nph/docs, accessed 08 May 2006)
8. Tonnesen H, Fugleholm AM, Jorgensen SJ. Evidence for health promotion in Hospitals. In: Groene O, Garcia-Barbero M, eds. Health promotion in hospitals. Evidence and quality management. Copenhagen, WHO Regional Office for Europe, 2005: 22-47 (http://www.euro.who.int/document/E86220.pdf accessed 08 May 2006).
9. Mckee M, Healy J, eds. Hospitals in a changing Europe. Oxford, Open University Press, 2001
10. Aiken LH, Clarke SP, Sloane DM, Sochalski J, Silber JH. Hospital nurse staffing and patient mortality, nurse burnout, and job dissatisfaction. JAMA. 2002 Oct 23-30;288(16):1987-93.
11. Ogden J. Health Psychology: A Textbook. Oxford, Open University Press, 1996.
12. Sabaté E. Adherence to Long-Term Therapies. Geneva, World Health Organization, 2003. (http://whqlibdoc.who.int/publications/2003/9241545992.pdf, accessed 08 May 2006.)

Hospitals consume a wide range of goods and produce high amounts of waste and hazardous substances. Introducing health promotion strategies can help to reduce the pollution of the environment and support the purchasing of locally produced, healthy products and produce.

Finally, as research and teaching institutions, hospitals produce, accumulate and disseminate a great body of knowledge and can have an impact on the local health structures and influence professional practice elsewhere.

3.2. Conceptualization of health promotion

The term health promotion is often not clearly distinguished from terms such as disease prevention, health education or empowerment[13]. The scope of disease prevention has been defined in the Health Promotion Glossary as "measures not only to prevent the occurrence of disease, such as risk factor reduction, but also to arrest its progress and reduce its consequences once established". The same source defines the scope of health education as comprising "consciously constructed opportunities for learning involving some form of communication designed to improve health literacy, including improving knowledge, and developing life skills which are conducive to individual and community health". Empowerment and health promotion refer to the broader concept laid out in the WHO Ottawa Charter as "the process of enabling people to increase control over, and improve, their health"[14].

In practice, these terms are frequently used complementarily or interchangeably and measures for their implementation may overlap, however, there are major conceptual differences with regard to the focus and impact of health promotion actions (Figure 1):

Figure 1: Strategies for health promotion[15]

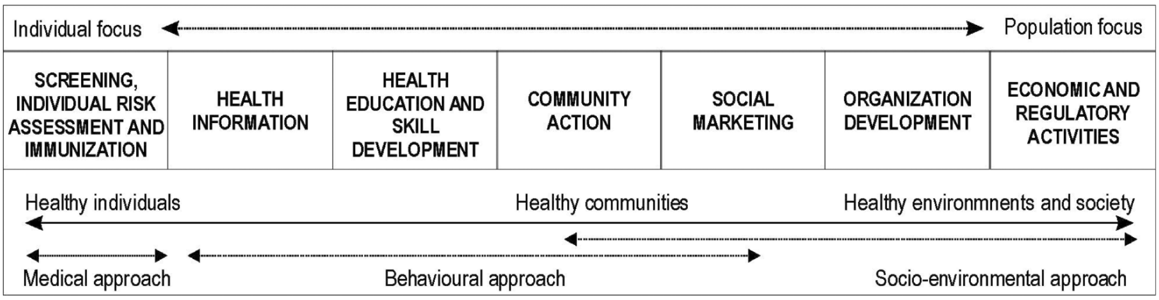

13. For full definitions and further references please see the Glossary in Section 7.
14. Ottawa Charter for Heatlh Promotion. Geneva , World Health Organization, 1986.
15. Bensber M. What are health promoting emergency departments? Melbourne, Department of Human Services, Victoria State Government, 2000.

Whereas the medical approach is directed at physiological risk factors (e.g. high blood pressure, immunization status), the behavioural approach is directed at lifestyle factors (e.g. smoking, physical inactivity) and the socio-environmental approach is directed at general conditions (such as unemployment, low education or poverty). Health promotion consequently includes, but goes far beyond medical approaches directed at curing individuals. While we acknowledge different theoretical schools behind the use of these terms, this manual only refers to health promotion activities, understood to cover both concrete actions such as assessing patients and providing specific information supporting the recognition of symptoms of disease as well as complex interventions such as supporting the patient to play an active role in the management of his/her condition. Excluded from this manual are activities such as screening and immunization, for which other quality criteria exist.

We are aware that the standards presented here do not cover the whole spectrum of activities associated with the WHO International Network of Health Promoting Hospitals. Standards addressing improving community health and environmental issues in hospitals may be developed at a later stage. Furthermore, the standards in this document are not intended to cover the whole spectrum of quality of health care. Quality of care, defined as "the degree to which health services for individuals and populations increase the likelihood of desired health outcomes and are consistent with current professional knowledge"[16], can be divided into different quality dimensions, such as clinical care, patient-centeredness, staff-orientation, responsive governance or efficiency. The standards and indicators described in this document only deal with the health promotion dimension of the quality of health care. A multidimensional perspective of quality assessment is promoted by the performance-assessment tool for quality improvement in hospitals, PATH[17].

3.3. Internal and external quality assessment

Approaches for quality assessment can be grouped broadly into internal and external assessment. Internal assessment refers to assessment based on judgement or institutional self-assessment based on standards. External assessment refers to expert inspection or accreditation[18]. Standards and performance indicators can be used for both internal assessment and continuous monitoring over time or for external assessment and comparative analysis of performance (such as benchmarking). Self-assessment and accreditation are the most common forms of internal and external assessment, respectively.

16. Glossary. European Observatory on Health Systems and Policies. (http://www.euro.who.int/observatory/glossary/toppage , accessed 08 May 2006).
17. Measuring hospital performance to improve the quality of care in Europe: a need for clarifying the concepts and defining the main dimensions. Report on a WHO Workshop, WHO Regional Office for Europe (http://www.euro.who.int/document/e78873.pdf , accessed 09 May 2006).
18. Shaw CD. External quality mechanisms for health care: summary of the EXPERT project. International journal for quality in health care , 2000, 3: 169-175

Self-assessment is a process used by healthcare organisations to accurately assess their level of performance in relation to established standards and to implement actions for continuous improvement. Self-assessment may cover all the hospital's activities or it may focus on specific issues, such as health promotion. It enables staff to identify areas of good practices and areas where there is a need for improvement. Hospital staff can then prioritize and plan the actions needed or replicate good practices in other departments of the hospital.

Accreditation is also usually based on self-assessment, but is followed up by an external peer evaluation process. The external assessment typically results in an overall evaluation of hospital quality, in identifying priority areas for improvement and, given that the required level of performance is achieved, in a formal declaration of the hospital being accredited. In many countries accreditation entails important legal, financial and marketing issues.

Evidence shows that self-assessment contributes to the goals of quality assurance in many ways. It is a low cost method driving changes in individual behaviour so as to increase compliance with standards, clarifies areas for improvement, gives participants ownership and can improve the communication between supervisors and subordinates.[19]

A main question regarding self-assessment is whether self-raters judge themselves accurately. A learning experience from self-assessment procedures is that well-performing hospitals are usually more critical than those hospitals that are not doing so well. Hospitals that adopt a culture of continuous quality improvement are more sensitive to their improvement potentials than those institutions that lack such culture[20]. For example, a study on self-assessment has shown that in the context of medication errors and quality of teamwork, well-performing teams recorded more errors than poor teams. To explore this further, team leaders were interviewed, and it was found that authoritarian dictatorial leaders led the poorer teams, who reported, perhaps not surprisingly, fewer errors. Self-assessment processes are thus prone to a range of biases that should be taken into consideration in the interpretation of results.

There are two main lessons to be learned through the process of self-assessment: quality improvement requires data on performance and a culture of improvement. Without data on performance, measured by standards or indicators, no clear direction for quality improvement can be recommended. And, without a culture of participation and support, even if data on the quality of care are available, quality improvement proposals cannot be implemented. Unfortunately, a lot of data is often collected without being useful or used for quality improvement initiatives. In that

19. Bose S, Oliveras E, Edson WN. How can self-assessment improve the quality of healthcare? Operations Research Issue Paper 2(4). Published for the U.S. Agency for International Development (USAID) by the Quality Assurance (QA) Project, Bethesda, MD and JHPIEGO Corporation, Baltimore, MD. 2001.
20. Edmondson AC. Learning from mistakes is easier said than done: group and organizational influences on the detection and correction of human error. Journal of Applied Behavioural Sciences, 1996. 32: 5-28

case, the data collection deviates time and other resources from other actions, impacting negatively on the quality of care.

The strategy of self-assessment is one of encouragement and education, assisting health care organizations as they develop their continuous quality improvement processes. It is not the intention of WHO to use the standards and indicators to formally assess, accredit or rank individual hospitals that are members of the Health Promoting Hospitals Network. The main purpose of developing the standards is to provide a tool that can support hospitals in assessing and improving health promotion activities. However, hospitals, in particular those in the Health Promoting Hospitals Network, are strongly encouraged to use the self-assessment tool. Since the standards are considered public domain, we further encourage all quality agencies and accreditation bodies to include the standards for health promotion in hospitals in their existing standards.

3.4. Standards for health promotion

In many countries quality agencies have developed standards for the quality of hospital care. Prominent agencies are present for example in: Australia, the Australian Council on Healthcare Standards (ACHS); Canada, the Canadian Council on Health Services Accreditation (CCHSA); France, the Haute Autorité de Santé (HAS); the United Kingdom, the Health Quality Services (HQS); and United States of America, the Joint Commission on Accreditation of Health Care Organizations (JCAHO)[21]. In addition, international standards were developed for example by the International Organization for Standardization and the international branch of the JCAHO, Joint Commission International (JCI). While most of these agencies work towards the achievement of a set of standards, other quality organizations, such as the European Foundation for Quality Management, are based on the principle of striving for excellence[22].

A review of the standards developed by the major accreditation agencies yielded that there was little reference to health promotion activities[23]. The WHO health promotion standards were therefore developed complementary to existing accreditation standards in line with the general philosophy of continuous quality improvement in organizations. In general:

Although standards are widely used in central medical units (e.g. laboratories, radiology, endoscopy, ambulance, operating theatres), they are not universally accepted by health professionals due to their lack of patient orientation and clinical outcomes.

21. Quality and accreditation in health care services. A global review. ISQuA and WHO. Geneva, Word Health Organization, 2003. (EIO/OSD/2003) (http://whqlibdoc.who.int/hq/2003/WHO_EIP_OSD_2003.1.pdf , accessed 08 May 2006).
22. Möller J. The EFQM excellence model. German experiences with the EFQM approach in health care. International Journal for Quality in Health Care, 2001, 13 (1): 45-49
23. Developing standards for Health Promotion in Hospitals. Background paper for the 1st Workshop on Standards for Health Promotion in Hospitals. Copenhagen, WHO Regional Office for Europe, 2002 (5038045/5).

Excellence models are typically built on organizational standards and when applied to health care lack standards on patient care processes. In view of the strong customer and staff orientation implied in some models, the WHO health promotion standards could be seen as a necessary supplement.

Accreditation standards have been applied to hospitals in many countries and partly address some components of patient education, though they lack an orientation towards the health of staff. The WHO standards can complement this approach towards a stronger orientation on the role of the patient in chronic care management and towards improving the health of staff.

Content of standards

The standards take into consideration the health potential of individuals and stress the importance of activating them through information, motivation, counselling, training or other activities to realize their health potential. Since information, education and advice only result in sustained behavioural change if supported by prevailing norms, rules and cultures, health promotion interventions in organizations have to address these underlying factors. Based on the philosophy of the Network of Health Promoting Hospitals (HPH), the standards not only address patient care but also the health of staff, links of the hospital to the community and organizational development. The content of the standards was defined based on the philosophy of the HPH network, the evidence-base for health promotion activities in hospitals and evidence on the implementation of quality management in organizations. The standards apply to all sectors of the health care organization directly or indirectly involved with patient care, e.g. public and private hospitals, and rehabilitation units, and can also be applied to psychiatric or pediatric hospitals.

The standards represent quality goals of the hospital organization related to three different perspectives: the clinical perspective, the patients' perspective and the organization/management perspective (Figure 2):

Figure 2: Clinical, patients' and management perspective

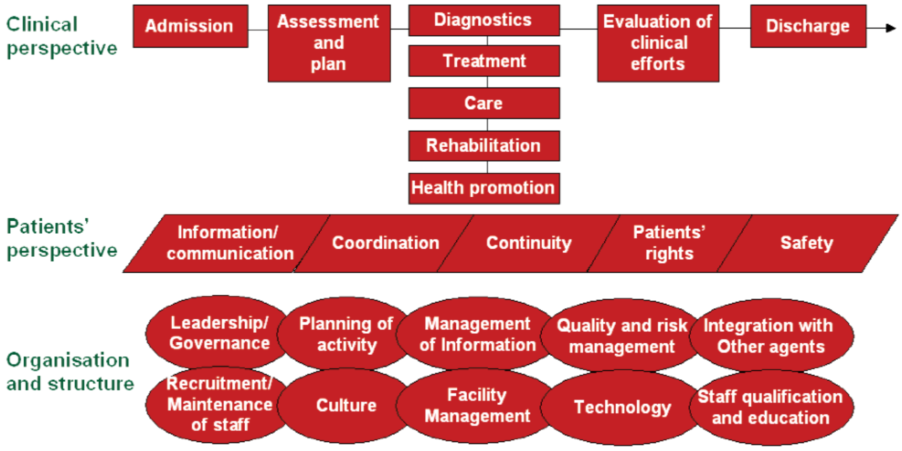

Development process

Following the suggestion of a working group under the Danish Network of Health Promoting Hospitals, a WHO expert working group was set up to develop the health promotion standards. The five standards describe the principles and actions that should be part of care in every hospital[24]. They were developed in accordance with the international requirements of the ALPHA programme, which include critical review of the literature, proposal for standards, review of standards, drafting preliminary standards, pilot testing, developing "final" standards, implementation and continuous revision and adjustment to changes in evidence and health care delivery[25].

A literature review proved that existing standards only marginally address the issue of health promotion in hospitals. After reaching agreement on the five dimensions the standards needed to address, a set of sub-standards was developed to operationalize the normative content of the standards. Substandards are based on the best evidence for health promotion actions. Measurable elements were further derived to improve the validity and reliability of the assessment procedure. They need to be documented against specific evidence for the assessment (e.g. if the substandard for ' identified responsibilities for assessment and implementation of health promotion' is evaluated positive, it has to be documented through e.g. a report specifying these responsibilities). The aim of measurable elements and evidence is hence to reduce the possible bias individuals may bring into the assessment procedure.

Measurable elements are assessed as 'yes, partly or no', but the importance of adding qualitative information to this assessment is underlined through a textbox below each measurable element that allows to document evidence, e.g. to elaborate why the measurable element was only assessed as partly or no, or to add any information that will be important for the development of the quality improvement plan. While an external evaluation is usually satisfied upon meeting a certain standard, it is more important for internal quality improvement to raise knowledge on how the quality can be improved further. The qualitative information contained in the textbox supports this goal.

The standards, sub-standards and measurable elements were piloted in 36 hospitals in nine European countries[26]. The main purpose of the pilot test was to evaluate whether they were perceived to be relevant and applicable by health professionals in different types of hospitals in European countries. In addition, the current fulfilment of the standards was assessed in order to identify their possible impact. At the same time

24. Developing standards for health promotion in hospitals. Copenhagen, WHO Regional Office for Europe, 2003 (http://www.euro.who.int/Document/IHB/hphstandardsfinrpt.pdf , accessed 08 May 2006).
25. International Standards for Health Care Accreditation Bodies. Internacional Society for Quality in Health Care (http://www.isqua.org/isquaPages/Accreditation/ISQuaIAPPrinciplesV2.pdf, accessed 08 May 2006).
26. Groene O, Jorgensen SJ, Fugleholm AM, Moeller L, Garcia-Barbero M. Results of a pilot test of standards for health promotion in nine European countries. International Journal for Quality Assurance in Health Care, 2005, 18 (4): 300-307 (www.emeraldinsight.com/0952-6862.htm , accessed 08 may 2006).

the preliminary standards were sent to international accreditation agencies and other organizations involved in quality improvement in health care for comments and suggestions.

Introduction to the standards

Each standard has three levels (Figure 3):

Level one is the level of the **standard** itself**.** The five standards address management policy; patient assessment, -information and -intervention; promoting a healthy workplace and continuity and cooperation.

Level two is the level of the **substandards**. Substandards operationalize the standard and break it down into its principle components. There are overall 24 substandards; the number of substandards per standards varies from 4 to 6.

Level three are the **measurable elements.** The measurable elements simply list what is required to be in full compliance with the standard. Listing the measurable elements is intended to provide greater clarity to the standards and help organizations educate staff about standards and prepare for the accreditation survey. Measurable elements are those requirements of the standard that will be reviewed and assessed to be not, partly or fully fulfilled. There are overall 40 measurable elements; the number of measurable elements per standard varies from 6 to 10.

Figure 3: Three level structure of standards for health promotion in hospitals

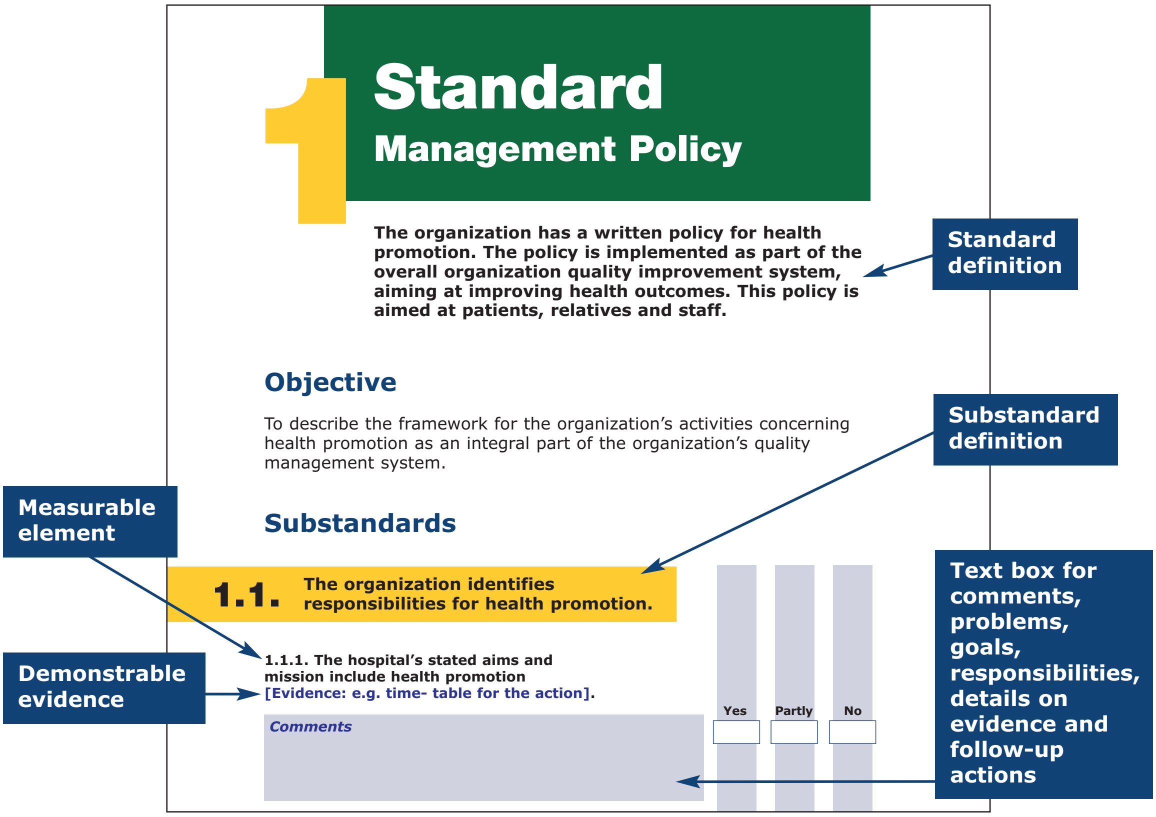

3.5. Indicators for health promotion

To complement the standards and provide a quantitative measurement tool to assess continuous improvement, performance indicators were developed. The indicators aim at filling the gap in assessing those outcomes that can be expected, if the structures and processes recommended in the formulation of standards are in place. In comparison to standards, indicators provide a *quantitative* basis for evaluating, monitoring and improving care and may assist health professionals and managers in developing targets and assessing progress on quality improvement activities.

Definition and characteristics of indicators

Indicators may serve very different purposes, e.g. to document quality of care, make comparisons over time between places, make judgements and set priorities, support accountability, regulation and/accreditation, support quality improvement, support patient choice over providers[27]. Indicators can thus be used for internal and/or external purposes. Internal purposes are related to the various management functions of the hospital as a health services delivery organization, such as a monitoring, evaluating and improving the functions in the long term (strategy) or short term. External purposes are related to accountability of stakeholders, such as the financing agency (either insurer or State), patients/consumers and the public at large.

Indicators address predominantly the process and outcome dimension of care, although they may also relate to structural characteristics (proportion of specialists to other doctors). They may be described in various ways, as a rate (number of events in a given population with a comparable denominator), proportion (percentage of events in a given population), ratio (relationship between two proportions), mean value (score from a survey) or absolute number. Indicators may also refer to sentinel events, i.e. phenomena that are intrinsically undesirable, where each event would trigger an in-depth investigation. An example for a sentinel event is wrong-side surgery. They can be generic (e.g. proportion of unscheduled returns to the operating room) or disease-specific (e.g. proportion of patients with myocardial infarction who receive a beta-blocker within < 24 hours after admission).
Within this self-assessment tool for health promotion in hospitals, indicators provide a quantitative measurement to assess progress over time for selected key processes and outcomes of selected health promotion indicators. They are meant to support continuous quality management internally and not to support any accountability decisions. However, they may be used in the future for benchmarking of health promotion activities, after they have proven to be scientifically sound and useful for the assessment and improvement of health promotion activities.

27. Mainz J. Defining and classifying clinical indicators for quality improvement. International Journal for Quality in Health care, 2003, 15 (6): 523-530.

Relating indicators to performance

An indicator provides information about a specific issue, while a set of indicators can provide information on a complex phenomenon (e.g. quality of care) which is not itself easily captured. Their validity lies in the strength of the relationship between the measurable element and the underlying concept. Indicators are only measurements and not judgments. A single indicator cannot be used to judge a hospital's performance, but a number of converging indicators may do so. Additional information such as reference points or explanatory variables, need to be taken into account to infer a judgment from an indicator. For instance, is an average length of stay for stroke of 10 days considered good or bad? Answer to this question depends on the average length of stay in the country, on the availability of rehabilitation beds, or the severity of the condition of the stroke patients in the hospital, on health status on discharge, on support provided by the hospital or organized by the hospital after return home, etc.

Using indicators for benchmarking

Indicators may be used for comparing performance between hospitals. A strong effort must be made to elaborate indicators, obtain clear definitions (numerator and denominator) and adjust for factors that may confound the comparison. A comparison of mortality rates for example would not be sound without adjusting the results for age, severity and co-morbidities. However, even after comprehensive risk-adjustment using multivariable statistical techniques, unexplained residual variation may remain that limit the power of the comparison.

A distinction needs to be drawn between simple comparisons and the concept of benchmarking. While *comparison* is the more loose term of relating measures to each other, *benchmarking* implies comparing the result of organizations' evaluations to the results of others and examining processes against those of others recognized as excellent, as a means of making improvements. Benchmarking hence implies comparing, identification of excellence and finally an examination of the factors that made an organization excel in the issue considered, compared to others.

Developing health promotion indicators

Common performance assessment frameworks to identify health promotion-related indicators currently in use were reviewed and only a few indicators were relevant to health promotion - most of the 300 indicators identified addressed the clinical effectiveness domain[28]. Therefore indicators were developed to complement the self-assessment tool for health promotion in hospitals, using working group and consensus methods. Two international meetings were held, bringing together experts

28. Champagne F, Guisset AL. A review of the indicators currently used in performance evaluation systems. Background paper. Copenhagen, WHO Regional Office for Europe, 2003 (5038066/9).

in the field of indicator development, quality improvement, accreditation and health promotion[29].

The health promotion indicators identified may not be so scientifically sound as others that have been in use in clinical practice for a long time. It should be stressed that performance assessment of health promotion activities in health care is still a new field and will benefit from further developments. In terms of data collection many of the health promotion indicators may require additional efforts, since the information is not generally captured through routine information systems. Nevertheless, this also entails the opportunity to gather more detailed data to allow better adjustments for comparisons or to better fit own organizational priorities.

The following indicators were selected to complement the five standards for health promotion presented in the self-assessment tool for health promotion in hospitals (Table 1).

Table 1: Overview on health promotion indicators

Standard/Domain	Indicator
Management policy	% of staff aware of health promotion policy
	% of patients aware of standards for health promotion
	% of budget dedicated to staff Health Promotion activities
Patient assessment	% of patients assessed for generic risk factors
	% of patients assessed for disease specific risk factors according to guidelines
	Score on survey of patients' satisfaction with assessment procedure
Patient information and intervention	% of patients educated about specific actions in self-management of their condition
	% of patients educated about risk factor modification and disease treatment options in the management of their conditions
	Score on survey of patients' experience with information and intervention procedures
Promoting a healthy workplace	% of staff smoking
	Smoking cessation
	Score of survey of staff experience with working conditions
	% of short term absence
	% of work-related injuries
	Score on burnout scale
Continuity and cooperation	% of discharge summaries sent to general practitioner or referral clinic within two weeks or handed to patient on discharge
	Readmission rate for ambulatory care sensitive conditions within 5 days
	Score on patient discharge preparation survey

29. Standards for health promotion: development of indicators for a self-assessment tool. WHO Regional Office for Europe, 2003. (http://www.euro.who.int/document/E84988.pdf , accessed 08 May 2006).

These indicators are described in more detail in section 7. For each indicator a descriptive sheet was prepared, addressing the following issues: domain, name, rationale and justification, numerator, denominator, data sources, stratification and notes.

3.6. Complementary use of standards and indicators

Standards focus mainly on structure and process whereas indicators mainly refer to process and outcome. Indicators have different metric properties and require a numerator and denominator. Standards need a clear definition but cannot necessarily be expressed in numeric terms. The relation between standards and indicators is complementary; they follow different philosophies and can - combined – support quality improvement activities in hospitals.

Using standards and indicators to assess quality

Standards-based evaluation is based on an assessment of whether appropriate structures, systems and processes are in place and functioning to achieve consistently favourable outcomes. The underlying assumption is that if standards are met, all the elements are in place to maximize the chances of good results (or outcomes) for patients, staff, or the public in general. Standards measure the efforts to foster quality. Questions hence raised through the assessment of standards compliance are "Is the organization doing the *right thing"* and "Is it *doing the right thing* consistently?". However, assessing standards does not yield information on the actual performance of an organization.

Performance assessment based on indicators, on the other hand, is a measure of what was done and how well it was done. Although a performance measure can be a structural characteristic or process, ideally performance measures address outcomes such as health outcomes, health status, patient satisfaction and resource use associated with care. The use of performance measures is limited by the challenge to collect and analyse complex data that need to be adjusted for possible confounding factors. Single indicators are difficult to interpret and it is rather the interrelationship of selected indicators that reflect quality improvement potentials.

According to Donabedian's typology, quality can be assessed through structure, process and outcomes. Although it is ultimately the outcome that matters, from the quality improvement perspective, it is important to study the link between structure, process and outcomes. The combination of complementary standards and indicators allows addressing the link between structures, processes and outcomes. Non-conformity with standards could provide a hint on structures or process changes that might be needed to maximize chances of positive outcomes. For example, information on negative outcomes such as sentinel events is important to monitor, but once the incident occurs, it is too late to change it.

Use of standards and indicators in the self-assessment tool

In the WHO self-assessment tool the standards are assessed by the measurable elements only. Health promotion indicators are meant to complement the assessment of standards' compliance reflecting sustained compliance with standards. It is up to the hospital to decide for which of the complementary indicators data should be collected, but at least one indicator for each domain should be collected. The self-assessment tool also contains a section on additional indicators where locally important or routinely available indicators may be reported. If a hospital chooses to add any additional indicators, it should be kept in mind that they should be described in the same way as those indicators contained in this manual.

The main use of indicators in the self-assessment procedure is to establish an operational baseline for further quality improvement actions that should be considered in the development of the quality improvement action plan. Such baseline will provide strong support for repeated measurements of performance over time.

4. Project implementation

4.1. Clarifying responsibilities

It is important to underline that health promotion cannot be delegated to a specific role or function within the hospital, it is 'everyone's responsibility in a hospital', and patients and each member of staff can contribute. As a quality issue health promotion activities should be assessed by standards and indicators just as other clinical quality issues. In this context it can be seen as 'added value' to existing quality initiatives, and the self-assessment of health promotion activities should be integrated into the existing hospital quality management system.

It is important to underline that all staff need to be committed to the success of the project. Commitment will vary according to interest and motivation, but advocacy to the project throughout the hospital and ownership are the two key pillars in the success. A team needs to be established for the project with clearly defined roles and responsibilities:

Partners:

Hospital management: Essential to the success of this project is the commitment of the chief executive, governing body and senior managers of the hospital, to ensure implementation of the action plan and to provide resources to undertake the task.

Project leader: It is also crucial that a project leader within the hospital is appointed to lead the process and train other staff in carrying out the self-assessment. Ideally, this person may already be responsible for other quality initiatives in the hospital as the project needs to be run as any other quality improvement activity.

Lead person for health promotion domains: The project leader may wish to nominate a lead person for each of the five health promotion domains (but lead persons may be responsible for more than one standard). Lead persons will need to take responsibility for assessing the level of compliance with the standard and substandards. They will be responsible for collecting the evidence that supports their response. They will also be responsible, in collaboration with other members of the steering group, to collect data for health promotion indicators.

Multidisciplinary steering group: The project leader needs to establish a multidisciplinary steering group that represents the staff at all levels. The steering group will need to meet on a regular basis to discuss progress with the self-assessment, generate ideas across disciplines and promote greater ownership of the project. Each hospital will have to identify the members of the steering group according to their

organization. Nevertheless, it is suggested that the following staff should be involved in the multidisciplinary steering group:

– a senior nurse who may also be responsible for quality /clinical audit,

– a senior and junior doctor,

– a senior manager,

– a human resources/personnel member,

– a member of staff from ancillary professions allied to medicine (e.g. physiotherapy, occupational therapy), general support medical services (e.g. radiology) and/or a member of staff from general non-clinical services.

4.2. Collecting data

Staff at different levels in the hospital should be involved in collecting data and supporting a collective response to the compliance of the standard. It is important to stress that there is very little value in one person completing the self-assessment without the involvement of relevant staff, as this may prevent staff from feeling ownership and therefore being involved in the learning process.

Three main data sources can be used within the hospital for the assessment of standards and indicators: routine information system, survey methods and audit procedures. In addition, some data may be provided by external partners, such as insurance companies that may have data on the health behaviour or absenteeism rate of staff.

Routine information systems may include information for some of the health promotion standards and indicators; useful in particular would be an electronic system for patient records that allows retrieving information on particular health promotion needs assessment or activities. Administrative databases may to some extent contain information on the workplace-health promotion indicators. Wherever data are available from routine sources, they should be used for the self-assessment in order to reduce the workload for data collection. However, the type of information contained in such databases may not be sufficiently specific for the purpose of assessing health promotion issues. In addition, there may be little flexibility to adapt it for other purposes.

A **survey** needs to be carried out for a range of health promotion indicators. This may be a survey on the experience of patients, but also a survey on the experience of staff members. As surveys should only be carried out using valid and reliable methods that may be resources intensive, the use of surveys should be restricted as much as possible. Alternatively, it should be considered to incorporate the items required to assess health promotion standards and indicators in existing patient and staff surveys.

An **audit** of clinical records is required in order to assess some of the standards addressing patient assessment, information and intervention. Details for audit procedures are summarized below.

Standards

The standards covering the management level and those covering all parts of the hospital (management policy, promoting a healthy workplace, continuity and cooperation) should be assessed by hospital management or the quality committee, if it exists. The standards for clinical activities (patient assessment, information and intervention) should be assessed at the level of clinical units. It is recommended, that 50 records for patients who are discharged and have been admitted to the unit within 3 months are chosen randomly for assessment. The audit group should be an interdisciplinary group of professionals with good knowledge about the documentation routines of the unit. The term “patients’ records” covers all kinds of documentation (medical record, nursing record, therapists and dieticians notes etc.) that need to be taken in consideration in the assessment of the hospital’s compliance with the standards.

Indicators

Indicators need to be reported in the self-assessment tool. However, the process of data collection to construct the indicators needs to be carried out separately. It is up to the hospital to decide which indicator they will choose, however, at least one indicator to complement each of the five standards should be collected.

Indicators need to be reported in the self-assessment tool for developing an action plan based on the assessment of both compliance with the standards, and the level of performance as per the indicators. Repeated measurements of indicators over time are necessary in order to reflect changes in the indicator. It is suggested that data on indicators be gathered every six months.

4.3. Interpreting results

Quality measures require summarizing data about the health care given to patients and expressing the results as a rate, ratio, frequency, distribution, or score for average performance.

Measures are often composed of a number and unit of measure with the number providing the magnitude and the unit providing the context for interpreting the number. It is difficult to interpret the result of a quality measure as good or poor, unless there is a standard of comparison by which it can be compared. The different types of comparisons are external comparison to similar providers at a single point in time, external comparison to similar providers over time, internal comparison over time (comparing scores before and after quality improvement efforts), and prescriptive standard (e.g. goals set by the regional health plan).

Well-established standards of care exist for some areas of health care treatment and services. In these cases, it is possible to conclude that a quality problem does or does not exist. Where there is no standard of care, results can be meaningful if compared to set goals such as those set by the organization implementing the measure or national goals. It is suggested to discuss the results of the self-assessment not only within the hospital, but also with other hospitals that carried out a self-assessment of health promotion activities.

4.4. Developing a quality improvement plan

In implementing this project it is recommended to follow the plan-do-check-act (PDCA) cycle. The PDCA cycle was originally conceived by Walter Shewhart in 1930s, and later adopted by W. Edwards Deming. The model provides a framework for the improvement of a process or system. It can be used to guide the entire improvement project, or to develop specific projects once target improvement areas have been identified. The PDCA cycle is designed to be used as a dynamic model (Figure 4). The completion of one turn of the cycle flows into the beginning of the next. Following in the spirit of continuous quality improvement, the process can always be re-analysed and a new test of change can begin.

Figure 4: Plan-Do-Check-Act Cycle

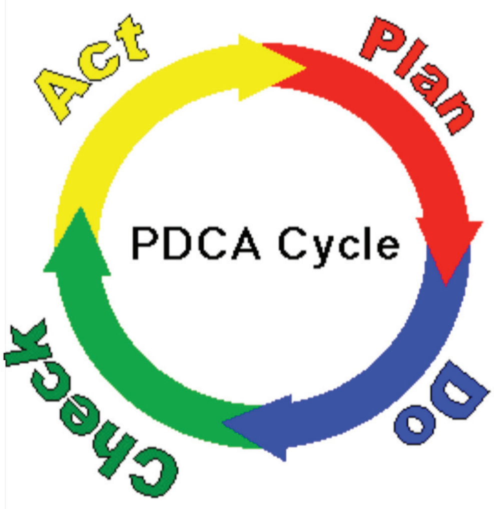

Plan: planning an activity, project or procedure aiming at improvement. This entails analysing what you intend to improve, looking for areas that hold opportunities for change and deciding where the greatest return on investment can be realized.

Do: carry out the change or test (preferably on a small scale) and implement the change you decided on in the plan phase.

Check: review results and analyse failure and success. This is a crucial step in the PDCA cycle. After you have implemented the change for a

short time, you must determine how well it is working. Is it really leading to improvement in the way you had hoped? You must decide on several measures with which you can monitor the level of improvement.

Act: Adopt the change, abandon it, or run through the cycle again.

In the self-assessment procedures, each section of the tool contains a text box where quality improvement actions, identified through the assessment of standards and indicators, need to be documented and responsibilities for that action need to be identified. Furthermore, it is required to document the timeframe for that action and its expected results. The final section of the self-assessment tool contains another textbox where overall quality improvement activities, and actions related to specific standards will be reported.

It is the responsibility of the project leader, together with the multidisciplinary steering group, to fill in the data as accurately as possible and at the same to be realistic about possible quality improvement actions in order to receive the top management's support for the implementation of the proposal. When the self-assessment is completed, the steering group will be able to identify areas of good practice and areas for development, where the hospital is not meeting the standards or substandards. An action plan can then be developed to address those issues. It is important that actions in the plan relate to local and national priorities or targets and the hospital's own available resources. The action plan should also be integrated into the existing management system of the hospital to monitor development.

After successful identification of quality improvement potentials, planning and implementation of activities, subsequent self-assessments need to be carried out to continue the quality improvement circle. As each full PDCA cycle comes to completion, a new and slightly more complex project can be undertaken to continuously improve services further.

5. Self-assessment forms

Responsibilities for the self-assessment

Responsibilities for the self-assessment should be documented in this section. One person has to take the overall responsibility (project leader). Additional responsibilities may be distributed for the various standards, according to the hospital's structure and human resources available (e.g. responsibility for the assessment of standards 1 and 5 may be with a senior management membrer, while responsibilities for the assessment of other standards may be with a member of clinical services). Each member should sign an agreement to confirm that they will collect, or supervise the collection of data.

The action plan should be discussed and planned by the whole steering group. The proyect leader approves the action plan and facilitates its implementation. The action plan needs to be presented to management.

Project leader

(Takes responsibility to overlook the overall self-assessment process and for the results presented)

**Name**	
**Function**	
**Date**	**/ /**

Signature

Members of the steering group

Name	Department	Title/ Function	Profession/ Discipline

Project leader for Standard 1: Management Policy

**Name**	
**Function**	
**Date**	/ /

Signature

Project leader for Standard 2: Patient Assessment

**Name**	
**Function**	
**Date**	/ /

Signature

Project leader for Standard 3: Patient Information and Intervention

**Name**	
**Function**	
**Date**	/ /

Signature

Project leader for Standard 4: Promoting a Healthy Workplace

**Name**	
**Function**	
**Date**	/ /

Signature

Project leader for Standard 5: Continuity and Cooperation

**Name**	
**Function**	
**Date**	/ /

Signature

1 Standard
Management Policy

The organization has a written policy for health promotion. The policy is implemented as part of the overall organization quality improvement system, aiming at improving health outcomes. This policy is aimed at patients, relatives and staff.

Objective

To describe the framework for the organization's activities concerning health promotion as an integral part of the organization's quality management system.

Substandards

1.1. The organization identifies responsibilities for health promotion.

1.1.1. The hospital's stated aims and mission include health promotion [Evidence: e.g. time- table for the action].

Yes ☐ Partly ☐ No ☐

Comments

1.1.2. Minutes of the governing body reaffirm agreement within the past year to participate in the WHO HPH project [Evidence: e.g. date for the decision or for payment of the annual fee].

Yes ☐ Partly ☐ No ☐

Comments

1.1.3. The hospital's current quality and business plans include health promotion (HP) for patients, staff and the community [Evidence: e.g. health promotion is explicit in the plan of action].

Yes	Partly	No

Comments

1.1.4. The hospital identifies personnel and functions for the coordination of HP [Evidence: e.g. staff member nominated for the coordination of HP].

Yes	Partly	No

Comments

1.2. The organization allocates resources for the implementation of health promotion.

1.2.1. There is an identifiable budget for HP services and materials [Evidence: e.g. budget or staff resources].

Yes	Partly	No

Comments

1.2.2. Operational procedures such as clinical practice guidelines or pathways incorporating HP actions are available in clinical departments [Evidence: e.g. check guidelines].

Yes	Partly	No

Comments

1.2.3. Specific structures and facilities required for health promotion (including resources, space, equipment) can be identified [Evidence: e.g. facilities to lift patients available].

Yes	Partly	No

Comments

1.3. The organization ensures the availability of procedures for collection and evaluation of data in order to monitor the quality of health promotion activities.

1.3.1. Data are routinely captured on HP interventions and available to staff for evaluation [Evidence: e.g. availability assessed in staff survey].

Yes	Partly	No

Comments

1.3.2. A programme for quality assessment of the health promoting activities is established [Evidence: e.g. time schedule for surveys is available].

Yes	Partly	No

Comments

Complementary indicators

__________ % of staff aware of health promotion policy

__________ % of patients (and relatives) aware of standards for health promotion

__________ % budget dedicated to staff HP activities

Additional indicators

(local indicators you may want to consider for the action plan)

Standard 1: Management Policy

Action plan

	Action	Responsible	Timeframe	Expected result
**General remarks**				
**1.1.**				
**1.2.**				
**1.3.**				

2 Standard
Patient Assessment

The organization ensures that health professionals, in partnership with patients, systematically assess needs for health promotion activities.

Objective

To support patient treatment, improve prognosis and to promote the health and well-being of patients.

Substandards

2.1. The organization ensures the availability of procedures for all patients to assess their need for health promotion.

2.1.1. Guidelines on how to identify smoking status, alcohol consumption, nutritional status, psycho-social-economic status are present [Evidence: e.g. check availability].

Yes	Partly	No

Comments

2.1.2. Guidelines/procedures have been revised within the last year [Evidence: e.g. check date, person responsible for revising guidelines].

Yes	Partly	No

Comments

2.1.3. Guidelines are present on how to identify needs for HP for groups of patients (e.g. asthma patients, diabetes patients, chronic obstructive pulmonary disease, surgery, rehabilitation) [Evidence: e.g. for groups of patients specifically treated in the clinical department].

Yes	Partly	No

Comments

2.2. The assessment of a patient's need for health promotion is done at first contact with the hospital. This is kept under review and adjusted as necessary according to changes in the patient's clinical condition or on request.

2.2.1. The assessment is documented in the patient's record at admission [Evidence: e.g. identified by patient records audit].

Yes	Partly	No

Comments

2.2.2. There are guidelines / procedures for reassessing needs at discharge or end of a given intervention [Evidence: e.g. guidelines present].

Yes	Partly	No

Comments

2.3. The patient's needs-assessment reflects information provided by others and ensures sensitivity to social and cultural background.

2.3.1. Information from referring physician or other relevant sources is available in the patient's record [Evidence: for all patients referred from physician].

Yes	Partly	No

Comments

2.3.2. The patient's record documents social and cultural background as appropriate [Evidence: religion that requires special diet or other specific attention. Social conditions indicating that the patient is at risk].

Yes	Partly	No

Comments

Standard 2 Patient Assessment:

Complementary indicators

_________ % of patients assessed for generic risk factors

_________ % of patients assessed for disease specific risk factors according to guidelines.

_________ score on survey of patients' satisfaction with assessment procedure

Additional indicators

(local indicators you may want to consider for the action plan)

Action plan

	Action	Responsible	Timeframe	Expected result
**General remarks**				
**2.1.**				
**2.2.**				
**2.3.**				

3 Standard
Patient Information and Intervention

The organization provides patients with information on significant factors concerning their disease or health condition and health promotion interventions are established in all patient pathways.

Objective

To ensure that the patient is informed about planned activities, to empower the patient in an active partnership in planned activities and to facilitate integration of health promotion activities in all patient pathways.

Substandards

3.1. **Based on the health promotion needs assessment, the patient is informed of factors impacting on their health and, in partnership with the patient, a plan for relevant activities for health promotion is agreed.**

3.1.1. Information given to the patient is recorded in the patient's record [Evidence: e.g. random review of patient records for all patients].

Yes	Partly	No

Comments

3.1.2. Health promotion activities and expected results are documented and evaluated in the records [Evidence: e.g. patient records' audit]

Yes	Partly	No

Comments

3.1.3. Patient satisfaction assessment of the information given is performed and the results are integrated into the quality management system [Evidence: e.g. various assessment methods: survey, focused group interview, questionnaire. Time schedule].

Yes | Partly | No

Comments

3.2. The organization ensures that all patients, staff and visitors have access to general information on factors influencing health.

3.2.1. General health information is available [Evidence: e.g. availability of printed or online information, or special information desk].

Yes | Partly | No

Comments

3.2.2. Detailed information about high/risk diseases is available [Evidence: e.g. availability of printed or online information, or special information desk].

Yes | Partly | No

Comments

3.2.3. Information is available on patient organizations [Evidence: e.g. contact-address is provided].

Yes | Partly | No

Comments

Standard 3 Patient Information and Intervention

Indicators

________ % of patients educated about specific actions in self-management of their condition

________ % of patients educated about risk factor modification and disease treatment options in the management of their condition

________ Score on survey of patients' experience with information and intervention procedures

Additional indicators

(local indicators you may want to consider for the action plan)

Standard 3: Patient Information and Intervention

Action plan

	Action	Responsible	Timeframe	Expected result
**General remarks**				
**3.1.**				
**3.2.**				

4 Standard
Promoting a Healthy Workplace

The management establishes conditions for the development of the hospital as a healthy workplace.

Objective

To support the development of a healthy and safe workplace, and to support health promotion activities of staff.

Substandards

4.1. The organization ensures the development and implementation of a healthy and safe workplace.

4.1.1. Working conditions comply with national/regional directives and indicators [Evidence: e.g. national and international (EU) regulations are recognized].

Yes ☐ Partly ☐ No ☐

Comments

4.1.2. Staff comply with health and safety requirements and all workplace risks are identified [Evidence: e.g. check data on occupational injuries].

Yes ☐ Partly ☐ No ☐

Comments

4.2. The organization ensures the development and implementation of a comprehensive Human Resources Strategy that includes training and development of health promotion skills of staff.

4.2.1. New staff receive an induction training that addresses the hospital's health promotion policy [Evidence: e.g. interviews with new staff].

Yes	Partly	No

Comments

4.2.2. Staff in all departments are aware of the content of the organization's health promotion policy [Evidence: e.g. annual performance evaluation or staff participation in the HP programme].

Yes	Partly	No

Comments

4.2.3. A performance appraisal system and continuing professional development including health promotion exists [Evidence: e.g. documented by review of staff files or interview].

Yes	Partly	No

Comments

4.2.4. Working practices (procedures and guidelines) are developed by multidisciplinary teams [Evidence: e.g. check procedures, check with staff].

Yes	Partly	No

Comments

4.2.5. Staff are involved in hospital policy-making, audit and review [Evidence: check with staff; check minutes of working groups for participation of staff representatives].

Yes	Partly	No

Comments

4.3. The organization ensures availability of procedures to develop and maintain staff awareness on health issues.

4.3.1. Policies for awareness on health issues are available for staff [Evidence: e.g. check for policies on smoking, alcohol, substance misuse and physical activity].

Yes	Partly	No

Comments

4.3.2. Smoking cessation programmes are offered [e.g. Evidence on availability of programmes].

Yes	Partly	No

Comments

4.3.3. Annual staff surveys are carried out including an assessment of individual behaviour, knowledge on supportive services/policies, and use of supportive seminars [Evidence: check questionnaire used for and results of staff survey].

Yes	Partly	No

Comments

Complementary indicators

- _________ **% of staff smoking**
- _________ **Smoking cessation**
- _________ **Score of survey of staff experience with working conditions**
- _________ **% of short-term absence**
- _________ **% of work-related injuries**
- _________ **Score on burnout scale**

Additional indicators

(local indicators you may want to consider for the action plan)

Action plan

	Action	Responsible	Timeframe	Expected result
**General remarks**				
**4.1.**				
**4.2.**				
**4.3.**				

5 Standard
Continuity and cooperation

The organization has a planned approach to collaboration with other health service providers and other institutions and sectors on an ongoing basis.

Objective

To ensure collaboration with relevant providers and to initiate partnerships to optimize the integration of health promotion activities in patient pathways.

Substandards

5.1. The organization ensures that health promotion services are coherent with current provisions and regional health policy plans.

5.1.1. The management board is taking into account the regional health policy plan [Evidence: e.g. regulations and provisions identified and commented in minutes of the meeting of management board].

Yes	Partly	No

Comments

5.1.2. The management board can provide a list of health and social care providers working in partnership with the hospital [Evidence: e.g. check update of list].

Yes	Partly	No

Comments

5.1.3. The intra- and intersectoral collaboration with others is based on execution of the regional health policy plan [Evidence: e.g. check congruency].

Yes | Partly | No

Comments

5.1.4. There is a written plan for collaboration with partners to improve the patients' continuity of care [Evidence: e.g. criteria for admittance, plan for discharge].

Yes | Partly | No

Comments

5.2. The organization ensures the availability and implementation of health promotion activities and procedures during out-patient visits and after patient discharge.

5.2.1. Patients (and their families as appropriate) are given understandable follow-up instructions at out-patient consultation, referral or discharge [Evidence: e.g. patients' evaluation assessed in patient surveys].

Yes | Partly | No

Comments

5.2.2. There is an agreed upon procedure for information exchange practices between organizations for all relevant patient information [Evidence: e.g. check availability of procedure].

Yes | Partly | No

Comments

5.2.3. The receiving organization is given in timely manner a written summary of the patient's condition and health needs, and interventions provided by the referring organization [Evidence: e.g. availability of copy].

Yes	Partly	No

Comments

5.2.4. If appropriate, a plan for rehabilitation describing the role of the organization and the cooperating partners is documented in the patient's record [Evidence: e.g. review of records].

Yes	Partly	No

Comments

Complementary indicators

_________ % of discharge summaries sent to GP or referral clinic within two weeks or handed to patient on discharge

_________ Readmission rate for ambulatory care sensitive conditions within 5 days

_________ Score on patient discharge preparation survey

Additional indicators

(local indicators you may want to consider for the action plan)

Standard 5: Continuity and Cooperation

Action plan

	Action	Responsible	Timeframe	Expected result
**General remarks**				
**5.1.**				
**5.2.**				

Overall assessment of standards compliance

Section		Yes	Partly	No
1 Management Policy	Total:	9	9	9
2 Patient Assessment	Total:	7	7	7
3 Patient Information and Intervention	Total:	6	6	6
4 Promoting a Healthy Workplace	Total:	10	10	10
5 Continuity and Cooperation	Total:	8	8	8
	Overall:	40	40	40

Overall action plan
(add more pages for full report if necessary)

General actions

Actions related to the assessment of specific standards and indicators

Descriptive sheets

6. Descriptive sheets for indicators

A descriptive sheet was prepared for each proposed indicator.[30] The descriptive sheets contain an operational definition, the rationale and justification for use (burden, importance, prevalence, potential for improvement), data sources and stratification, validity and guide for interpretation.

It needs to be emphasized that some of the proposed indicators cannot be described as clinical indicators in terms of International Classification of Diseases (ICD) codes and clear in- and exclusion criteria, but rather rely on survey measures or audit procedures. The validity and reliability of some of the indicators is still limited compared to well-established clinical indicators, as indicators on health promotion activities in hospitals are to a large extent still under development. The descriptive sheets in the following pages need to be updated periodically to reflect new evidence and assessments of validity for these indicators. A comprehensive overview on clinical and other health care quality-related indicators is available online.[31]

Section 1. Rationale and description

This section gives a brief justification of why the indicator should be used. It is crucial to make clear what the indicator is supposed to measure, its strengths and limits. It is also extremely valuable that users understand why it is important to gather data on the indicator, in order to motivate them to accept the indicator, go through the burden of data collection, and ensure data quality.

Section 2: Operational definition

The objective of this section is to share a common language. Operational definitions are provided to support uniform data collection longitudinally and across hospitals and countries. If indicators are to be used for comparisons, operational definitions (and the underlying data) need to be largely standardized.

30. Some of the indicators chosen are congruent with those selected in the WHO Performance Assessment Tool for Quality Improvement in Hospitals (PATH). For those indicators we used the same definition. For further information on the PATH project, please see www.euro.who.int/ihb

31. National quality measures clearinghouse (www.qualitymeasures.ahrq.gov , accessed 08 May2006).

Section 3: Data source and stratification

In this section we provide some information on data collection issues. As it is not the primary objective of this project to perform comparative analysis between organizations, clear definitions and homogeneous data collection procedures are only important to improve reliability and validity of indicators for longitudinal analysis. For some indicators we provide information on which data to collect, where they are available, by whom they are collected, and what are the data quality control mechanisms.

Section 4. Interpretation guide

The last section provides information on how to use the indicator results. The objective of this project is to encourage reflection on current practices and initiating quality improvement activities based on the results of self-assessment. To this end indicators should not be simply considered as a statement of good or bad performance, nor should indicators be interpreted in isolation.

Table 2: Descriptive sheet for staff awareness of policy

**Domain**	**Management Policy**
Indicator 1	***% of staff aware of health promotion policy***
Rationale and description	It is the main aim of the corresponding standard that management develops a policy for health promotion targeted at staff, patients and relatives. Core components in this process are the definition of responsibilities, development of competences and identification of infrastructures.<br><br>Since it is not the objective is not to assess directly the compliance with standards and substandards but rather their sustained implementation, it could be considered that the awareness of staff about the policy and its contents is an indirect and reflective, but highly associated performance measure. Even if staff is aware but not satisfied by the policy, the measure is conclusive in emphasizing democratic and transparent working processes.
Numerator	Number of staff that can name the main components of the health promotion policy.
Denominator	All staff
Data source	Audit or survey methods. Many hospitals carry out repeated surveys on staff health and satisfaction, and items could be included to assess the awareness of staff about management's health promotion policy. Otherwise an ad hoc survey based on a convenience sample can be considered a reasonable measure to obtain data on this indicator.
Stratification	By departments, by professional groups
Notes/ interpretation	This indicator has not systematically been validated. However, similar indicators assessing the staff's awareness of the organization's guiding principles are available and have proven to be conclusive,[32] and a wealth of literature in the organizational sciences describing impact of staff involvement on organizational effectiveness is available[33]. A high awareness among staff members of the management policy is reflective of good communication between management and staff, itself an important issue that potentially triggers support for management decision making, building of shared identity and organizational learning processes.<br><br>On the other hand awareness alone does not ensure health promotion action among staff members, in particular if staff do not have resources to implement the policy. The indicator is thus useful for monitoring how management policies are communicated to staff members, it does not measure actual health promotion performance. After initial progression through the PDCA cycle, subsequent measures may address knowledge of staff membesr on specific content issues of the policy, and assessment of staff potential and resources to implement the policy.

32. Roberts KH. Managing high reliability organizations. Calif Manage Rev 1990, 32: 101-113.
33. Dierkes M, Antal AB, Child J, Nonaka I. Handbook of organizational learning and knowledge. Open University Press, 2001.

Table 3: Descriptive sheet for patients' (and relatives') awareness

Domain	**Management Policy**
Indicator 2	***% of patients (and relatives) aware of standards for health promotion***
Rationale and description	Similar to above, patients need to be aware of the health promotion policy in order to benefit the most from it. Patients who are informed about the policy are more likely to demand further information on their condition, on lifestyle changes and on other institutions, associations or self-help groups. The underlying assumption is that, the more empowered the patient is, the more likely he/she will request further information to understand his/her condition, the health care process and the implications for follow up.<br><br>There is strong evidence to support that better empowered patients have better health outcomes[34]. Likewise, this information should be to the avail of relatives; however, the burden of data collection may be higher since there are no systematic records of relatives visiting the hospital.
Numerator	Number of patients aware of the health promotion policy.
Denominator	All patients
Data source	Survey methods. In many countries, hospitals send satisfaction questionnaires after discharge to elicit the patients' views and experiences about the care provided. Such a survey can include an item on patients' awareness of the health promotion policy. Discharge interviews could also be used to assess in a convenience sample of patients to what extent they are aware of the policy.
Stratification	For the hospital: By department. For the patient: by age, sex and educational background.
Notes/ Interpretation	This indicator has not systematically been validated. However, there is strong research evidence on the link between empowerment and health. This indicator is useful for monitoring how health professionals communicate with the patient and whether they are able to explain what their hospital is doing in health promotion. After initial progression through the PDCA cycle, subsequent measures may address knowledge of patients on specific health promotion interventions they either received or they consider important. This information could be useful for initiating further health promotion activities.

34. McKee M. In: The evidence for health promotion effectiveness. Report for the European Commission by the International Union for Health Promotion and Education. Brussels, 2000.

Table 4: Descriptive sheet for percentage of health promotion budget

**Domain**	**Management Policy**
Indicator 3	***% of budget dedicated to staff HP activities***
Rationale and description	This indicator addresses direct financial resources available for health promotion-related training, meetings and infrastructures. There are little data available on the extent of health promotion activities within hospitals. A survey in a sample of more than 1400 companies in seven European countries indicate that "activities which might be regarded as coming from the health promotion arena (e.g. eating, alcohol or smoking policies) tend to take place rarely"[35].<br><br>Areas of health promotion activities can be grouped as follows: 1) health screening, 2) promoting healthy behaviour, 3) organizational interventions, 4) safety/physical environment, 5) social and welfare. Illustrations: worksite smoking cessation programs, stress counselling service, workplace childcare centre, influenza vaccine, alcohol dependence screening, etc.<br><br>The degree of freedom to allocate funds within hospitals varies greatly between countries and public/private status and the available total budget. It also depends on national policies and legislation on health promotion within the workplace. A potential adverse effect is that hospitals are evaluated merely on the budget for health promotion activities, and not on the volume and quality of their health promotion activities; they might as well just define a budget without being convinced of its usefulness nor without really ever using it.
Numerator	Budget for activities dedicated to staff health promotion
Denominator	Average number of employees on payroll during the period (alternative: average number of full time employees)
Data source	Financial data
Stratification	According to area of health promotion (see definitions above)
Notes/ Interpretation	This indicator has not been systematically evaluated. There is no evidence to support that defining a health promotion budget has an impact on extent and quality of health activities. However, even if the activities do not produce the expected results, their implementation can be viewed as a concern for staff health and hence a staff orientation.

35. European Foundation for the Improvement of Living and Working Condition (EFILWC) Workplace Health Promotion in Europe – Programme summary. Luxembourg, Office for Official Publications of the European Communities, ed., 1997: 40.

Table 5: Descriptive sheet for patients assessed for generic risk factors

**Domain**	**Patient assessment**
Indicator 4	***% of patients assessed for generic risk factors***
Rationale and description	The indicator measures whether patients were assessed for generic risk factors. Generic risk factors play a role in the development of many diseases; yet, they are frequently not assessed nor recorded in medical or nursing records. The purpose of the indicator is to support a systematic assessment of all patients for generic risk factors and document these in order to be available for other health professionals than those carrying out the assessment.
Numerator	Total number of patients with evidence in their records that they were assessed for risk factors, including smoking, nutrition, alcohol.
Denominator	Number of patients (in a random sample)
Data source	Clinical audit of medical or nursing records (sample)
Stratification	To be stratified by age.
Notes/ Interpretation	-

Table 6: Descriptive sheet for patients assess for specific risk factors

**Domain**	**Patient assessment**
Indicator 5	***% of patients assessed for disease specific risk factors according to guidelines***
Rationale and description	The indicator measures whether patients were assessed for risk factors against guidelines. Many hospital admissions for chronic conditions can be related to a few risk factors that are strongly involved in the development of the condition, e.g. smoking habits, excessive alcohol consumption, poor nutrition and lack of physical activity.<br><br>Hospitals frequently provide care to ameliorate the symptoms of the chronic condition without tackling the underlying risk factors. While it is not necessarily the responsibility of the hospital to provide e.g. intensive smoking cessation programmes, it should nevertheless a) provide the patient with information on where to obtain such services and b) feed back to the primary care physician the presence of the risk factors and its relation to the condition the patient was admitted for.
Numerator	Total number of patients with evidence in their records that they were assessed for risk factors against guidelines, including smoking, nutrition and alcohol.
Denominator	Number of patients (in a random sample)
Data source	Clinical audit of medical or nursing records (sample)
Stratification	To be stratified by age.
Notes/ Interpretation	The difference to indicator no 4 lies in its focus on specific diseases and the use of guidelines in the assessment process. The rationale is that for specific conditions concrete risk factors exist beyond the generic risk factors such as smoking and lack of physical activity.

Table 7: Descriptive sheet for patient satisfaction

**Domain**	**Patient assessment**
Indicator 6	***Score on survey of patients' satisfaction with assessment procedure***
Rationale	Patient satisfaction questionnaires are an accepted tool to assess the overall quality of care from the patient's perspective. Assessment is often carried out upon discharge or within a brief timeframe (e.g. two weeks) after discharge. Patient satisfaction questionnaires are a useful tool to assess the *overall* quality of care; while patients may not be able to assess technical components of the intervention for which they were admitted, they are best equipped to assess issues of care, very important for the patients, such as respect for privacy, continuity of care, confidentiality, the feeling that all their needs, including emotions, were taken care of. Patient satisfaction and patient experience questionnaires are a main tool to assess those aspects of care, which the Health Promoting Hospitals' projects aims to foster.
Numerator	Score on survey (e.g. patients being satisfied with care - depends on the use of the assessment tool; hospitals may choose their own cut-off point as to at which target they want to aim at).
Denominator	All patients
Data source	Survey
Stratification	By hospital department and by the patients' age, sex and educational background.
Notes	Often hospitals use surveys that were constructed in-house and may infer bias in the assessment of the patient's satisfaction or experience, although a number of survey tools are available online in various languages. We strongly recommend the use a standardized assessment tool that has undergone comprehensive psychometric validation. Examples are e.g. the Picker Questionnaire, ServQual or Consumer Health Plan Assessment.

Table 8: Descriptive sheet for patient education for self-management

**Domain**	**Patient information and intervention**
Indicator 7	***% of patients educated about specific actions in self-management of their condition***
Rationale and description	A high volume of care provided is for patients with chronic conditions. However, the hospital stay is only a small component in the care chain required by chronic patients. Other main components of care are provided outside the hospital in the ambulatory sector, or managed by the patient and their relatives themselves. In fact, the empowerment of the patient to take a more active role in his/her care is a main contribution towards improving the quality of care and reducing health system expenditure.<br><br>In order to involve patients more actively in the care process, it is a prerequisite to provide them with more information about their condition and about possible actions related to improving their condition. Better educated patients have shown to have fewer complications and readmissions and thus contribute to both quality of life and cost-containment[36, 37].
Numerator	Patients who can name actions in self-management of their condition
Denominator	All patients (sample)
Data source	Survey, interviews
Stratification	Departments, age, sex
Notes	The survey method should specify the main self-management action the patient has to be able to name.

36. Tattersall RL. The expert patient: a new approach to chronic disease management for the twenty-first century. Clinical Medicine, 2002, 2(3): 227-9.
37. Lorig K. et al. Evidence suggesting that a chronic disease self-management program can improve health status while reducing hospitalization: a randomized trial. Medical Care 1999, 37(1): 5-14.

Table 9: Descriptive sheet for patient risk factor education

**Domain**	***Patient information and intervention***
Indicator 8	***% of patients educated about risk factor modification and disease treatment options in the management of their conditions***
Rationale and description	Ditto indicator no 7. The difference is the focus on specific conditions
Numerator	Patients who can name actions in self-management of their condition
Denominator	Patients diagnosed with a specific condition (e.g. stroke, chronic obstructive pulmonary disease, myocardial infarction, diabetes mellitus)
Data source	Survey, interviews
Stratification	Department, age, sex, condition
Notes	The survey has to specify the main issues in risk factor modification and disease treatment options, for each condition that the patient has to be able to name.<br><br>The indicator is very similar to indicator no 7 and both may be collected simultaneously, followed by stratification by condition.

Table 10: Descriptive sheet for patients' information/intervention score

**Domain**	**Patient information and intervention**
Indicator 9	***Score on survey of patients' experience with information and intervention procedures***
Rationale and description	Ditto no 6. Questionnaires on patient experiences with care are an accepted tool to assess the overall quality of care from the patient's perspective.<br><br>In addition to indicator no 6 which assesses the global quality of care, this indicator assesses the experience with the process of information and interventions, e.g. did the physician provide information about the disease but in a manner incomprehensible to the patient?
Numerator	Score on survey (e.g. patients being satisfied with care - depends on the use of the assessment tool; hospitals may choose their own cut-off point as to which target they want to aim).
Denominator	All patients
Data source	Survey
Stratification	By hospital department and by the patients' age, sex and educational background.
Notes	Often hospitals use surveys constructed in-house which may infer bias in the assessment of the patient's satisfaction or experience, although a number of survey tools are available online in various languages. We strongly recommend the use of a standardized assessment tool that has undergone comprehensive psychometric validation. Examples are e.g. the Picker Questionnaire[38], ServQual[39] or Consumer Health Plan Assessment[40].

38. Jenkinson C, Coulter, A, Bruster S. The Picker patient experience questionnaire: development and validation using data from in-patient surveys in five countries. International Journal for Quality in Health Care, 2002, 14: 353-358.
39. Buttle F. SERVQUAL: review, critique, research agenda. European Journal of Marketing ,1996, 30 (1): 8-32
40. Hibbard JH, Slovik P, Jewett JJ. Informing consumer decisions in health care: implications from decision-making research. The Milbank Quarterly ,1997, 75(3): 395-414.

Table 11: Descriptive sheet for staff smoking

**Domain**	**Promoting a healthy workplace**
Indicator 10	***% of staff smoking***
Rationale and description	Health Promoting Hospitals have committed themselves to become a smoke-free setting, and hence the proportion of staff smoking is a single indicator reflective of the overall success of implementing health promotion in hospitals.<br><br>Smoking has indisputably a negative effect on health.Despite this, a large number of health professionals is still smoking[41, 42]. Staff smoking behaviour is further related to patients' compliance with lifestyle counselling: patients, who are admitted to the hospital with a condition related to their smoking habits, are more responsive to lifestyle counselling. However, receiving that advice by a health professional smoking him/herself limits the success of reducing smoking behaviour among patients.
Numerator	Number of staff smoking
Denominator	All staff
Data source	Survey
Stratification	By department, discipline, age and sex
Notes/ interpretation	The European Network of Smoke-free hospitals[43] developed a survey measure including 13 standard questions to be able to compare differences between hospitals in various European countries.

41. Fichtenberg CM, GLantz SA. Effect of smoke-free workplaces on smoking behaviour: systematic review. British Medical Journal, 2002, 325: 188
42. Moller AM, Villebro N, Pedersen T, Tonnesen H. Effect of preoperative smoking intervention on postoperative complications: a randomized clinical trial. Lancet 2002, 359: 114-117
43. European Network of Smoke-free hospitals (http://ensh.free.fr , accessed 08 May 2006).

Table 12: Descriptive sheet for smoking cessation

**Domain**	**Promoting a healthy workplace**
Indicator 11	***Smoking cessation: % of staff members who were either current smokers or recent quitters and who received advice to quit smoking.***
Rationale and description	Smoking has a significant impact on mortality from smoking-related diseases. Smoking cessation reduces the risk of premature death, and a high proportion of smokers are interested in stopping smoking completely. This measure addresses whether smokers and recent quitters, who were seen by a managed care organization practitioner during the measurement year, received advice to quit smoking. It has been shown that clinician advice to stop smoking improves cessation rates by 30%.[44]<br><br>This measure assesses the percentage of members 18 years and older who were continuously enrolled during the measurement year, who were either current smokers or recent quitters, who were seen by a managed care organization practitioner during the measurement year and who received advice to quit smoking.
Numerator	The number of members in the denominator who responded to the survey and indicated that they had received advice to quit smoking from a managed care organization practitioner during the measurement year
Denominator	The number of members who responded to the survey and indicated that they were either current smokers or recent quitters and that they had one or more visit(s) with a managed care organization practitioner during the measurement year.
Data source	Administrative data and patient survey search and Quality (AHRQ).
Stratification	Stratified by departments, profession, sex and age.
Notes/ interpretation	This is a standard indicator in HEDIS system. For detailed specifications regarding the National Committee on Quality Assurance (NCQA) measures, refer to *HEDIS Volume 2: Technical Specifications*, available from the NCQA Web site at www.ncqa.org.

44. National Quality Measures Clearinghouse (http://www.qualitymeasures.ahrq.gov/, accessed 08 May 2006).

Table 13: Descriptive sheet for staff experience

**Domain**	**Promoting a healthy workplace**
Indicator 12	***Score of survey of staff experience with working conditions***
Rationale and description	A range of instruments exists to assess staff experiences with working conditions. Results of job content questionnaire (measures psychological demands, job decision latitude and social support at work) are associated with both medically certified and non-certified sickness absences among nurses[45]. This indicator is strongly linked to indicator no 10 (satisfaction correlates negatively with absenteeism)
Numerator	Score on survey (e.g. staff being satisfied with working conditions - depends on the use of the assessment tool; hospitals may choose their own cut-off point as to at which target they want to aim).
Denominator	All staff
Data source	Survey
Stratification	By hospital department and by the patients' age, sex and educational background.
Notes	The survey may be chosen by the hospital, e.g. the Katasek job content questionnaire[46]. Information may also be already available from existing staff health surveys. However, it is recommended only using surveys or items that have proven their validity and reliability after psychometric validation.

45. Bourbonnais R, Mondor M. Job strain and sickness absence among nurses in the Province of Québec. American Journal of Industrial Medicine, 2001, 39:194-202.
46. Karasek R, BRisson C, Kawakami N et al. The job content questionnaire: an instrument for internationally comparative assessments of psychosocial job characteristics. Journal of Occupational Health Psychology, 1998, 3(4): 322-5.

Table 14: Descriptive sheet for short-term absenteeism

**Domain**	**Promoting a healthy workplace**
Indicator 13	***% of short term absence***
Rationale and description	Absenteeism has a high burden on hospital functioning: Cost to compensate for loss of working hours, increased workload for the remaining staff, lost productivity, lower quality if highly skilled personnel providing essential services cannot be replaced. Short-term absence is most disturbing because of its unpredictable nature and it allows less time to adjust schedule, to take steps to replace absent worker, etc. But absenteeism has also a positive impact: Short-term absenteeism can be an effective coping strategy in the presence of stressful conditions. "Working through" illness: Incidence of employees attending work despite being ill is increasing in CIS countries, mainly because of fear of dismissal or financial motivations (loss of earnings).[47]<br>In Europe, the absenteeism rate (including temporary and permanent work incapacity) ranges from 3.5% in Denmark to 8% in Portugal[48]<br>Different interventions may decrease absenteeism at hospital level: employee assistance programs, training and goal setting programs, policy changes to increase employees' accountability for their absence, scheduling changes such as flexible time, and games or token economies. Situational predictors of absenteeism such as organizational permissiveness, role problems, pay, and job characteristics are partly under the hospital's sphere of influence.[49]
Numerator	Number of days of medically or non-medically justified absence for seven days or less in a row (short-term absenteeism) or 30 days or more (long-term absenteeism), excluding holidays, among nurses and nurse assistants
Denominator	Total equivalent full time nurses and nurses assistants * number of contractual days per year for a full time staff member (e.g. 250 days)
Data source	Routine information system at hospital or departmental level or data from health insurance companies.
Stratification	Collect data by age, sex and qualification (nurse or assistant)
Notes/ interpretation	This indicator is measured only for nurses and nurses' assistants. Administrative and support staff and physicians are not included.<br>For long-term absenteeism, maternity leaves, including preventive leaves, are excluded. However, sick leave during pregnancy is included.

47. Arford CW. Failing health systems: Failing health workers in Eastern Europe. Report on the Basic Security Survey for the International Labour Office and Public Services International Affiliate in the Health Sector in Central and Eastern Europe. Geneva, International Labour Office, 2001 (Available on www.ilo.org/ses, accessed 08 May 2006).
48. European Foundation for the Improvement of Living and Working Conditions – European Foundation for the Improvement of Living and Working Conditions, 1997.
49. Dalton DR, William DT. Turnover, transfer, absenteeism: an independent perspective. Journal of Management, 1993, 19(2): 193-219.

Table 15: Descriptive sheet for work-related injuries

**Domain**	**Promoting a healthy workplace**
Indicator 14	***% of work-related injuries***
Rationale and description	There are great health risks for hospital staff such as the exposure to HIV and other bloodborne viruses (e.g. hepatitis B and C). The risk of transmission of hepatitis C virus from a needlestick injury is estimated to 1.8% - 3%. Early antiviral treatment of acute hepatitis C virus infection has high cure rates. Injuries have a sustained effect on worker anxiety and distress[50] and direct cost of medical follow-up for at-risk exposure.<br><br>In a meta-analysis of the literature, the mean rate of sharp injuries per 10.000 healthcare workers to bloodborne pathogen was equal to 4%, with surgeons being mostly affected[51]. Only 35% physicians adhered to universal precautions and non-compliance with universal precautions was and non-compliance was associated with a considerably increased risk of both MCE and PCE, especially in non-surgical specialties. Note: it is difficult to compare rates because of varying definitions and methods. The US General Accounting Office (GAO) estimated that 75% needlestick injuries were preventable by eliminating unnecessary use (25%), by using needles with safety features (29%), by using safer work practices (21%). Injuries are significantly associated with work environment characteristics (time pressure of work). In Laiken et al. (1997), working in hospitals characterized by professional nurse practice models and taking precautions to avoid blood contact was significantly associated with fewer injuries among nurses.
Numerator	Number of percutaneous injuries in one year (includes needlestick injuries and sharp devices injuries)
Denominator	Average number of full-time equivalent exposed staff (physician, nurses, phlebicist)
Data source	Survey among staff on self-reported injuries, further data: insurance claims, human resources specific register
Stratification	By profession, area of care (ICU, operating theatre, emergency, surgical, medical department), time on the day (or weekdays vs weekends), work experience
Notes	Alternatively the indicator could address all work-related injuries and then be stratified by type of injury.

50. Fisman DN, Mittelman MA, Sorock GS, Harris AD. Willingness to pay to avoid sharp-related injuries: a study in injuried health care workers. AJIC: American Journal of Infection Control, 2002, 30(5): 283-287.
51. Trim JC, Elliott TS. A review of sharps injuries and preventive strategies. Journal of Hospital Infection 2003, 53(4): 237-242.

Table 16: Descriptive sheet for burnout scale

**Domain**	**Promoting a healthy workplace**
Indicator 15	***Score on burnout scale***
Rationale and description	Burnout is a physical, mental, and emotional response to constant levels of high stress. Most cases are work-related. Burnout usually results in physical and mental fatigue, and can include feelings of hopelessness, powerlessness and failure. Burnout often arises from excessive demands that are either internally imposed (such as having very high expectations of yourself) or externally imposed (by family, job, or society) and is frequently associated with work situations in which a person feels overworked, under-appreciated, confused about expectations and priorities, given responsibilities that are not commensurate with pay, insecure about layoffs, and/or overcommitted with home and work responsibilities.[52] While stress is a "hurry sickness;" burnout represents a "depletion syndrome." These are very distinct concepts. Burnout is not simply excessive stress. Rather, it is a complex human reaction to stress, and it relates to feeling that your inner resources are inadequate for managing the tasks and situations presented.[53]<br><br>Burnout is caused by (among others): changes in the organization, the demands of your job, your supervisor, or the industry, changes in your interests or values pertaining to work, under-utilization of your abilities and skills, feeling trapped in a situation that provides little recognition and few rewards for work well done, being assigned more tasks than you can possibly handle, having no voice in regulating your assignments or working conditions or struggling with tasks that are beyond your ability. Results of staff burnout can be psychosomatic illnesses (psychological/emotional problems which manifest themselves physically), digestive problems, headaches, high blood pressure, heart attacks, teeth grinding and fatigue. Better hospital organization, work environments and management styles can reduce burnout among staff.[54]
Numerator	— Score on burnout inventory —
Denominator	— Score on burnout inventory —
Data source	Survey
Stratification	By departments, sex, professional group and age.
Notes/ interpretation	A controversial issue in the literature is whether client severity correlates positively with burnout or job dissatisfaction. In comparing different departments (internal medicine, oncology) severity may be controlled for, or at least, the impact of different patient groups and work conditions should be considered.

52. Cordes C., Dougherty TW. A review and integration of research on job burnout. Academy of Management Review, 1993, 18 (4): 621-656.
53. Collins MA. The relation of work stress, hardiness, and burnout among full-time hospital staff nurses. Journal for Nurses in Staff Development. 1996, 12(2): 81-5.
54. Schulz R, Greenley JR, Brown R. Organization, management, and client effects on staff burnout. Journal of Health and Social Behavior, 1995, 36(4): 333-45

Table 17: Descriptive sheet for discharge summaries

**Domain**	**Continuity and cooperation**
Indicator 16	***% of discharge summaries sent to GP or referral clinic within two weeks or handed to patient on discharge***
Rationale and description	Indicator of continuity of care. Chronic patients require continuous follow up care, however, in many contexts there is insufficient communication between the providers of health and socialfare. Fragmented delivery of care results in delays in the detection of complications, or declines in health status because of irregular or incomplete assessments, or inadequate follow-up; failures in self-management of the illness or risk factors as a result of patient passivity or ignorance, stemming from inadequate or inconsistent patient assessment, education, motivation, and feedback; reduced quality of care due to the omission of effective interventions or the commission of ineffective ones; undetected or inadequately managed psychosocial distress.<br><br>While this indicator does not cover the whole spectrum of continuity of care,[55] the burden of data collection is not too high and it reflects an important component of continuity of care: the information flow between secondary and primary care providers.<br><br>The indicator needs to be stratified by condition: the importance of discharge letters varies with the condition for which the patient was admitted. Further work may address where the discharge letter contains information on laboratory results that were produced in the hospital and required for the follow-up care provided by the primary care physician.
Numerator	Discharge letters sent to GP or handed to patient within two weeks after discharge
Denominator	All discharge letters
Data source	Administrative audit or survey
Stratification	By department or by professional.
Notes/ interpretation	Depending on whether data are available in routine information system, this indicator may cause a high work burden for the data collection. In some countries, discharge information may not be sent directly to ongoing care provider but is handed to the patient at discharge. While timeliness of discharge information is important, completeness or comprehension by the receiver is not assessed with this indicator. Subsequent quality improvement cycles may include an assessment of these issues.

55. For a review of measures of continuity of care see: Groene O. Approaches towards measuring the integration and continuity in the provision of health care services. In: Kyriopoulis, J, ed. Health systems in the world: From evidence to policy. Athens, Papazisis, 2005.

Table 18: Descriptive sheet for readmission rate

**Domain**	**Continuity and cooperation**
Indicator 17	***Readmission rate for ambulatory care sensitive conditions within 5 days***
Rationale and description	Readmissions reflect the impact of hospital care on the condition of the patient after discharge[56].The underlying assumption is that something providers did or left undone during the prior stay led to the need for re-hospitalization. It could be either due to sub-standard care during hospitalization, poor discharge preparation or follow-up.<br>To be considered as a readmission, four conditions must be met: 1) meeting certain diagnoses or procedure, 2) subsequent emergent or urgent admission (non elective), 3) time between the discharge after the initial episode and the admission for the subsequent hospitalization within a specified time period, 4) initial episode did not end with the patient signing himself out against medical advice (or died).<br>Other potential exclusion criteria: patients already receiving continuous care at a primary care clinic, chemotherapy or radiotherapy; residing in or planned to go to nursing home; admitted only to undergo a procedure. Asthma and diabetes are two ambulatory care sensitive conditions. For ambulatory care sensitive conditions, evidence suggests that admission could be avoided, at least in part, through better outpatient care. From 9% to 48% of all readmissions have been judged to be preventable through better patient education, pre-discharge assessment and domiciliary care.[57]<br><br>The hospital influence is limited as readmissions after medical stay often indicate the progression of the disease rather than discrete outcomes of care. By focussing on early readmissions and imposing more stringent ime frame for readmission, impact of natural progression of the disease and post-discharge care is limited.
Numerator	Total number of patients admitted through the emergency department after discharge –within a fixed follow-up period– from the same hospital and with a readmission diagnosis relevant to the initial care.
Denominator	Total number of patients admitted for selected tracer condition (e.g. asthma, diabetes, pneumonia, CABG)
Data source	Routine information systems and hospital clinical records. Reimbursement claims to purchasing agency.
Stratification	Adjusted by age, sex, severity. Since its is not the aim of the pilot implementation to facilitate benchmarking between hospitals, further adjustments are not necessary at this stage.
Notes	Exclusion: Patients who died during hospitalization or who were discharged to another acute care hospital are excluded.

56. Westert GP, Lagoe RJ, Keskimäki I, Leyland A, Murphy M. An international study of hospital readmissions and related utilization in Europe and the USA. Health Policy, 2002, 61: 262-278.
57. Benbassat J, Taragin M. Hospital readmissions as a measure of quality of health care. Archives of Internal Medicine, 2000, 160:1074-1081.

Table 19: Descriptive sheet for discharge preparation

**Domain**	**Continuity and cooperation**
Indicator 18	***Score on patient discharge preparation survey***
Rationale and description	Discharge preparation is particularly important for patients suffering from chronic conditions and in need of follow up care. Patients need to be able to understand their condition, be aware of risk factors and symptoms for remissions, need to understand the treatment options and drug regimes and follow up care plan. Many patients are not aware of these issues, thus having a major impact on the long-term quality of care and possible resulting in complications, readmissions and reduced quality of life.<br><br>This indicator is a measurement tool of how well an organization is preparing its patients for discharge. Various tools exist that were developed specifically for this purpose, and some existing questionnaires on patient satisfaction and experience include items on discharge preparation. It is recommended to use existing tools where available, or apply standardized and validated tools where not.<br><br>In case of adapting existing tools, items to be included are for example: "Can you name the condition you were admitted for?", "Can you name the symptoms of your condition?", "Do you feel confident that you understood how to take your medication", "Do you know whom to address in case your condition deteriorates?"
Numerator	— for this indicator a score need to be build based on a survey measure —
Denominator	— for this indicator a score need to be build based on a survey measure —
Data source	Survey
Stratification	By departments and patient characteristics (sex, age, condition)
Notes/ interpretation	Adjustment by department and patient characteristics may be important as perceived discharge preparation is influence by a range of factors.

Glossary

7. Glossary

The following glossary presents the main terms used in this manual group around major themes, such as:

Underlying concepts
Quality dimensions
Stakeholders
Assessment procedures/Data collection
Understanding measures
Interpreting results
Health promotion activities
Quality improvement actions

Terms were compiled from standard glossaries such as International Society for Quality in Health Care (ISQuA)[58], Joint Commission International (JCI)[59] and the European Observatory on Health Systems and Policies[60], etc.

Underlying concepts

Accountability
Responsibility and requirement to answer for tasks or activities. This responsibility may not be delegated and should be transparent.

Risk
Chance or possibility of danger, loss or injury. This can relate to the health and wellbeing of staff and the public, property, reputation, environment, organizational functioning, financial stability, market share and other things of value.

Health
Health is defined in the WHO constitution of 1948 as: A state of complete physical, social and mental wellbeing, and not merely the absence of disease or infirmity. Within the context of health promotion, health has been considered less as an abstract state and more as a means to an end, which can be expressed in functional terms as a resource which permits people to lead an individually, socially and economically productive life. Health is a resource for everyday life, not the object of living. It is a positive concept emphasizing social and personal resources as well as physical capabilities.

Culture
A shared system of values, beliefs and behaviours.

Ethics
Standards of conduct that are morally correct.

58. International Society for Quality in Health Care: http://www.isqua.org.au/isquaPages/Links.html (accessed 08 May 2006).
59. Joint Commission International http://www.jointcommission.org/ (accessed 08 May 2006).
60. European Observatory on Health Systems and Policies: http://www.euro.who.int/observatory/glossary/toppage (accessed 08 May 2006).

Rights
Something that can be claimed as justly, fairly, legally, or morally one's own. A formal description of the services that clients can expect and demand from an organization.

Values
Principles, beliefs or statements of philosophy that guide behaviour and that may involve social or ethical issues.

Vision
Description of what the organization would like to be.

Health development
Health development is the process of continuous, progressive improvement of the health status of individuals and groups in a population.

Reference: Terminology Information System. WHO, Geneva, 1997

The Jakarta Declaration describes health promotion as an essential element of health development.

Mission
A broad written statement in which the organization states what it does and why it exists. The mission sets apart one organization from another.

Need
Physical, mental, emotional, social or spiritual requirement for wellbeing. Needs may or may not be perceived or expressed by those in need. They must be distinguished from demands, which are expressed desires, not necessarily needs.

Philosophy
A statement of principles and beliefs made by the organization, by which it is managed and delivers services.

Quality dimensions

Quality
The degree of excellence, extent to which an organization meets clients' needs and exceeds their expectations.

Access
Ability of clients or potential clients to obtain required or available services when needed within an appropriate time.

Appropriateness
The degree to which service is consistent with a client's expressed requirements and is provided in accordance with current best practice.

Continuity
The provision of coordinated services within and across programs and organizations, and over time.

Cultural appropriateness
The design and delivery of services consistent with the cultural values of clients who use them.

Effectiveness
The degree to which services, interventions or actions are provided in accordance with current best practice in order to meet goals and achieve optimal results.

Efficiency
The degree to which resources are brought together to achieve results with minimal waste, re-work and effort.

Safety
The degree to which the potential risk and unintended results are avoided or minimised.

Stakeholders

Accreditation body
The organization responsible for the accreditation program and the granting of accreditation status.

Customers
The patients/clients of a client organization. Internal customers/staff of the organization.

Community
Collectivity of individuals, families, groups and organizations that interact with one another, cooperate in common activities, solve mutual concerns, usually in a geographic locality or environment.

Community
A specific group of people, often living in a defined geographical area, who share a common culture, values and norms, are arranged in a social structure according to relationships which the community has developed over a period of time. Members of a community gain their personal and social identity by sharing common beliefs, values and norms which have been developed by the community in the past and may be modified in the future. They exhibit some awareness of their identity as a group, and share common needs and a commitment to meeting them.

Governance
The function of determining the organization's direction, setting objectives and developing policy to guide the organization in achieving its mission, and monitoring the achievement of those objectives and the implementation of policy.

Governing body
Individuals, group or agency with ultimate authority and accountability for the overall strategic directions and modes of operation of the organization. Also known as the council, board, board of commissioners, etc.

Health professionals
Medical, nursing or allied health professional staff who provide clinical treatment and care to clients, having membership of the appropriate professional body and, where required, having completed and maintained registration or certification from a statutory authority.

Organization
Comprises all sites/locations under the governance of, and accountable to, the governing body/owner(s).

Partners
The organizations with which the organization works and collaborates to provide complementary services.

Partnerships
Formal or informal working relationships between organizations where services may be developed and provided jointly, or shared.

Staff
Employees of the organization.

Stakeholder
Individuals, organizations or groups that have an interest of share in services.

Assessment procedures/Data collection

Document control system
A planned system for controlling the release, change, and use of important documents within the organization, particularly policies and procedures. The system requires each document to have a unique identification, to show dates of issue and updates and authorization. Issue of documents in the organization is controlled and copies of all documents are readily traceable and obtainable.

Accreditation
A self-assessment and external peer assessment process used by health care organizations to accurately assess their level of performance in relation to established standards and to implement ways to continuously improve.

Assessment
Process by which the characteristics and needs of clients, groups or situations are evaluated or determined so that they can be addressed. The assessment forms the basis of a plan for services or action.

Audit
A systematic independent examination and review to determine whether actual activities and results comply with planned arrangements.

Competence
Guarantee that an individual's knowledge and skills are appropriate to the service provided and assurance that the knowledge and skill levels are regularly evaluated.

Complaint
Expression of a problem, an issue, or dissatisfaction with services that may be verbal or in writing.

Complementary
Services or components that fit with each other, or supplement one another, to form more complete services.

Confidentiality
Guaranteed limits on the use and distribution of information collected from individuals or organizations.

Consent
Voluntary agreement or approval given by a client.

Data
Unorganised facts from which information can be generated.

Evaluation
Assessment of the degree of success in meeting the goals and expected results (outcomes) of the organization, services, programmes or clients.

Evidence
Data and information used to make decisions. Evidence can be derived from research, experiential learning, indicator data, and evaluations. Evidence is used in a systematic way to evaluate options and make decisions.

Health outcomes
A change in the health status of an individual, group or population which is attributable to a planned intervention or series of interventions, regardless of whether such an intervention was intended to change health status.

Intermediate health outcomes
Health promotion outcomes

Information
Data which are organized, interpreted and used. Information may be in written, audio, video or photograph form.

Information systems
Systems for planning, organizing, analysing and controlling data and information, including both computer-based and manual systems.

Performance
The continuous process by which a manager and a staff member review the staff member's performance, set performance goals, and evaluate progress towards these goals.

Qualitative
Data and information expressed with descriptions and narratives, a method that investigates the experience of users through observation, interviews.

Quantitative
Data and information expressed in numbers and statistics, a method that investigates phenomena with measures.

Reliability
Extent to which results are consistent through repeated measures by different measurers, or at different times by the same measurer, when what is measured has not changed in the interval between measurements.

Research
Contribution to an existing body of knowledge through investigation, aimed at the discovery and interpretation of facts.

Validity
Extent to which a measure truly measures only what it is intended to measure.

Results (Outcomes)
The consequences of a service.

Quality assessment
Planned and systematic collection and analysis of data about a service, usually focused on service content and delivery specifications and client outcomes.

Survey
External peer assessment which measures the performance of the organization against an agreed set of standards.

Surveyor
External peer reviewer, assessor of organizational performance against agreed standards.

Licensure
Process by which a government authority grants permission to an individual or health care organization to operate, or to an individual practitioner, to engage in an occupation or profession.

Peer assessment
A process whereby the performance of an organization, individuals or groups is evaluated by members of similar organizations, or the same profession or discipline and status as those delivering the services.

Personnel record
Collection of information about a staff member covering personnel issues such as leave, references, performance appraisals, qualifications, registration, and employment terms.

Understanding measures

Scope
The range and type of services offered by the organization and any conditions or limits to service coverage.

Services
Products of the organization delivered to clients, or units of the organization that deliver products to clients.

Standard
A desired and achievable level of performance against which actual performance is measured.

Criteria
Specific steps to be taken, or activities to be done, to reach a decision or a standard.

Procedures
Written sets of instructions conveying the approved and recommended steps for a particular act or series of acts.

Policies
Written statements which act as guidelines and reflect the position and values of the organization on a given subject.

Measurable elements
Measurable elements of a standard are those requirements of the standard and its intent statement that will be reviewed and assigned a score during the accreditation survey process. The measurable elements simply list what is required to be in full compliance with the standard. Each element is already reflected in the standard or intent statement. Listing the measurable elements is intended to provide greater clarity to the standards and help organizations educate staff about standards and prepare for the accreditation survey (JCI International Standards, 2003).

Indicator
Performance measurement tool, screen or flag that is used as a guide to monitor, evaluate, and improve the quality of services. Indicators relate to structure, process, and outcomes.

Interpreting results

Benchmarking
Comparing the results of organizations' evaluations to the results of other interventions, programmes, or organizations, and examining processes against those of others recognised as excellent, as a means of making improvements.

Best practice
An approach that has been shown to produce superior results, selected by a systematic process, and judged as exemplary, or demonstrated as successful. It is then adapted to fit a particular organization.

Health promotion

Disease prevention
Disease prevention covers measures not only to prevent the occurrence of disease, such as risk factor reduction, but also to arrest its progress and reduce its consequences once established. Reference: adapted from the Glossary of Terms used in the Health for All series. WHO, Geneva, 1984

Primary prevention is directed towards preventing the initial occurrence of a disorder. Secondary and tertiary prevention seeks to arrest or retard existing disease and its effects through early detection and appropriate treatment; or to reduce the occurrence of relapses and the establishment of chronic conditions through, for example, effective rehabilitation. Disease prevention is sometimes used as a complementary term alongside health promotion. Although there is frequent overlap between the content and strategies, disease prevention is defined separately. Disease prevention in this context is considered to be action which usually emanates from the health sector, dealing with individuals and populations identified as exhibiting identifiable risk factors, often associated with different risk behaviours.

Education
Systematic instruction and learning activities to develop or bring about change in knowledge, attitudes, values or skills.

Empowerment for health
In health promotion, empowerment is a process through which people gain greater control over decisions and actions affecting their health.
Empowerment may be a social, cultural, psychological or political process through which individuals and social groups are able to express their needs, present their concerns, devise strategies for involvement in decision-making, and achieve political, social and cultural action to meet those needs.

Enabling
In health promotion, enabling means taking action in partnership with individuals or groups to empower them, through the mobilization of human and material resources, to promote and protect their health.

Health behaviour
Any activity undertaken by an individual, regardless of actual or perceived health status, for the purpose of promoting, protecting or maintaining health, whether or not such behaviour is objectively effective towards that end.

Health communication
Health communication is a key strategy to inform the public about health concerns and to maintain important health issues on the public health agenda. The use of the mass and multimedia and other technological innovations to disseminate useful health information to the public, increases awareness of specific aspects of individual and collective health as well as importance of health in development. Reference: adapted from Communication, Education and Participation: A Framework and Guide to Action. WHO (AMRO/PAHO), Washington, 1996

Health education
Health education comprises consciously constructed opportunities for learning involving some form of communication designed to improve health literacy, including improving knowledge, and developing life skills which are conducive to individual and community health.
Health education is not only concerned with the communication of information, but also with fostering the motivation, skills and confidence (self-efficacy) necessary to take action to improve health. Health education includes the communication of information concerning the underlying social, economic and environmental conditions impacting on health, as well as individual risk factors and risk behaviours, and use of the health care system. Thus, health education may involve the communication of information, and development of skills which demonstrates the political feasibility and organizational possibilities of various forms of action to address social, economic and environmental determinants of health.

Health literacy
Health literacy represents the cognitive and social skills which determine the motivation and ability of individuals to gain access to, understand and use information in ways which promote and maintain good health.
Health literacy implies the achievement of a level of knowledge, personal skills and confidence to take action to improve personal and community health by changing personal lifestyles and living conditions. Thus, health literacy means more than being able to read pamphlets and make appointments. By improving people's access to health information, and their capacity to use it effectively, health literacy is critical to empowerment. Health literacy is itself dependent upon more general levels of literacy. Poor literacy can affect people's health directly by limiting their personal, social and cultural development, as well as hindering the development of health literacy.

Health promoting hospitals
A health promoting hospital does not only provide high quality comprehensive medical and nursing services, but also develops a corporate identity that embraces the aims of health promotion, develops a health promoting organizational structure and culture, including active, participatory roles for patients and all members of staff, develops itself into a health promoting physical environment and actively cooperates with its community. Reference: based on the Budapest Declaration on Health Promoting Hospitals. WHO, Regional Office for Europe, Copenhagen, 1991

Health promotion
Health promotion is the process of enabling people to increase control over, and to improve their health. Reference: Ottawa Charter for Health Promotion. WHO, Geneva,1986 Health promotion represents a comprehensive social and political process, it not only embraces actions directed at strengthening the skills and capabilities of individuals, but also action directed towards changing social, environmental and economic conditions so as to alleviate their impact on public and individual health. Health promotion is the process of enabling people to increase control over the determinants of health and thereby improve their health. Participation is essential to sustain health promotion action.

Intersectoral collaboration
A recognized relationship between part or parts of different sectors of society which has been formed to take action on an issue to achieve health outcomes, or intermediate health outcomes, in a way which is more effective, efficient or sustainable than might be achieved by the health sector acting alone.

Life skills
Life skills are abilities for adaptive and positive behaviour, that enable individuals to deal effectively with the demands and challenges of everyday life.

Lifestyle (lifestyles conducive to health)
Lifestyle is a way of living based on identifiable patterns of behaviour which are determined by the interplay between an individual's personal characteristics, social interactions, and socioeconomic and environmental living conditions.

Re-orienting health services
Health services re-orientation is characterized by a more explicit concern for the achievement of population health outcomes in the ways in which the health system is organized and funded. This must lead to a change of attitude and organization of health services, which focuses on the needs of the individual as a whole person, balanced against the needs of population groups.

Quality improvement actions

Follow-up
Processes and actions taken after a service has been completed.

Goals
Broad statements that describe the outcomes an organization is seeking and which provide direction for day-to-day decisions and activities. The goals support the mission of the organization.

Guidelines
Principles guiding or directing action.

Capacities
Abilities, resources, assets, and strengths of groups or individuals to deal with situations and meet their needs.

Contract
Formal agreement that stipulates the terms and conditions for services that are obtained from, or provided to, another organization. The contract and the contracted services are monitored and coordinated by the organization and comply with the standards of the government and the organization.

Coordination
The process of working together effectively with collaboration among providers, organizations and services in and outside the organization to avoid duplication, gaps, or breaks.

Leadership
Ability to provide direction and cope with change It involves establishing a vision, developing strategies for producing the changes needed to implement the vision; aligning people; and motivating and inspiring people to overcome obstacles.

Management
Setting targets or goals for the future through planning and budgeting, establishing processes for achieving those targets and allocating resources to accomplish those plans. Ensuring that plans are achieved by organizing, staffing, controlling and problem-solving.

Objective
A target that must be reached if the organization is to achieve its goals. It is the translation of the goals into specific, concrete terms against which results can be measured.

Operational plan
The design of strategies, which includes the processes, actions and resources to achieve the goals and objectives of the organization.

Quality activities
Activities which measure performance, identify opportunities for improvement in the delivery of services, and include action and follow-up.

Quality control
The monitoring of output to check if it conforms to specifications or requirements and action taken to rectify the output. It ensures safety, transfer of accurate information, accuracy of procedures and reproducibility.

Quality improvement
Ongoing response to quality assessment data about a service in ways that improve the processes by which services are provided to clients.

Quality plan
The current action plan for meeting service quality requirements.

Quality project
A timebound quality improvement plan for an identified service or area.

Risk management
A systematic process of identifying, assessing and taking action to prevent or manage clinical, administrative, property and, occupational health and safety risks in the organization.

Strategic plan
A formalised plan that establishes the organization's overall goals, and that seeks to position the organization in terms of its environment

The WHO Regional Office for Europe

The World Health Organization (WHO) is a specialized agency of the United Nations created in 1948 with the primary responsibility for international health matters and public health. The WHO Regional Office for Europe is one of six regional offices throughout the world, each with its own programme geared to the particular health conditions of the countries it serves.

Member States

Albania
Andorra
Armenia
Austria
Azerbaijan
Belarus
Belgium
Bosnia and Herzegovina
Bulgaria
Croatia
Cyprus
Czech Republic
Denmark
Estonia
Finland
France
Georgia
Germany
Greece
Hungary
Iceland
Ireland
Israel
Italy
Kazakhstan
Kyrgyzstan
Latvia
Lithuania
Luxembourg
Malta
Monaco
Netherlands
Norway
Poland
Portugal
Republic of Moldova
Romania
Russian Federation
San Marino
Serbia and Montenegro
Slovakia
Slovenia
Spain
Sweden
Switzerland
Tajikistan
The former Yugoslav Republic of Macedonia
Turkey
Turkmenistan
Ukraine
United Kingdom
Uzbekistan

WHOLIS number: E88584
Original: EUR/06/5064214

World Health Organization
Regional Office for Europe
Scherfigsvej 8, DK-2100 Copenhagen Ø, Denmark
Tel.: +45 39 17 17 17. Fax: +45 39 17 18 18
E-mail: postmaster@euro.who.int
Web site: www.euro.who.int
